# Serum biomarker trajectory clusters predict functional outcome and quality of life for traumatic brain injury

**DOI:** 10.1093/braincomms/fcag055

**Published:** 2026-02-23

**Authors:** Thanh Son Do, Chantal Carnes, Zhihui Yang, Firas Kobeissy, Hamad Yadikar, Gayla Olbricht, Olli Tenovuo, Jussi P Posti, Ewout W Steyerberg, Lindsay Wilson, Nicole von Steinbüchel, Endre Czeiter, Andras Buki, David K Menon, Andrew I R Maas, Kevin K Wang, Tayo Obafemi-Ajayi

**Affiliations:** Department of Computer Science, Missouri State University, Springfield, MO 65897, USA; Pathology Department, Meharry Medical College, Nashville, TN 37208, USA; Department of Emergency Medicine, University of Florida, Gainesville, FL 32611, USA; Department of Emergency Medicine, University of Florida, Gainesville, FL 32611, USA; Department of Neurobiology, Center for Neurotrauma, Multiomics & Biomarkers, the Neuroscience Institute, Morehouse School of Medicine, Atlanta, GA 30310, USA; Center for Visual and Neurocognitive Rehabilitation, Atlanta VA Health Care System, Decatur, GA 30033, USA; Department of Biological Sciences, Faculty of Science, Kuwait University, Sabah Al-Salem University City, P.O. Box 5969, Safat 13060, Kuwait; Mathematics & Statistics Department, Missouri University of Science & Technology, Rolla, MO 65409, USA; Clinical Neurosciences, University of Turku, 20520 Turku, Finland; Neurocenter, Department Neurosurgery and Turku Brain Injury Center, Turku University Hospital, 20520 Turku, Finland; Clinical Neurosciences, University of Turku, 20520 Turku, Finland; Neurocenter, Department Neurosurgery and Turku Brain Injury Center, Turku University Hospital, 20520 Turku, Finland; Julius Center for Health Sciences and Primary Care, University Medical Center Utrecht, Universiteitsweg 100, 3584 CG Utrecht, the Netherlands; University of Stirling, Scotland, UK; Institute of Medical Psychology and Medical Sociology, University Medical Center Göttingen, Georg-August-University, Göttingen 37073, Germany; Department of Neurosurgery, Medical School, Molecular Medicine Research Group, Szentágothai Research Centre, HUN-REN-PTE Clinical Neuroscience MR Research Group, University of Pécs, H-7623 Pécs, Hungary; Department of Neurosurgery, Örebro University, 70 182 Örebro, Sweden; Department of Medicine, University of Cambridge, Cambridge, UK; Department of Neurosurgery, Antwerp University Hospital, 2650 Edegem, Belgium; Department of Translational Neuroscience, Faculty of Medicine and Health Science, University of Antwerp, 2610 Wilrijk, Belgium; Department of Emergency Medicine, University of Florida, Gainesville, FL 32611, USA; Department of Neurobiology, Center for Neurotrauma, Multiomics & Biomarkers, the Neuroscience Institute, Morehouse School of Medicine, Atlanta, GA 30310, USA; Center for Visual and Neurocognitive Rehabilitation, Atlanta VA Health Care System, Decatur, GA 30033, USA; Engineering Program, Missouri State University, Springfield, MO 65897, USA

**Keywords:** blood-based biomarkers, ensemble cluster validation model, traumatic brain injury prognosis, machine learning

## Abstract

Serum brain-enriched biomarkers are increasingly employed in the clinical evaluation of traumatic brain injury (TBI) to assist with triage, neuroimaging decisions, and prognostication. However, the potential of temporal biomarker trajectories to inform disease monitoring and long-term outcomes remains underexplored. We aim to identify distinct biomarker trajectory (TRAJ) profiles in traumatic brain injury patients and to examine their associations with long-term clinical outcomes. The study included 373, CT-positive Intensive Care Unit (ICU) traumatic brain injury patients (256 with initial Glasgow Coma Scale 3–12) from the Collaborative European NeuroTrauma Effectiveness Research in TBI (CENTER-TBI) core study who had at least two serum samples collected between days 1 and 5 post-injury. Six biomarkers -glial fibrillary acidic protein, ubiquitin C-terminal hydrolase-L1, neurofilament light chain, Tau, S100B, and neuron-specific enolase- were analysed. Optimal cluster solutions were determined using a composite validation index derived from seven internal clustering metrics. Distinct high and low trajectory classes emerged for all biomarkers; each comprising at least 40% of the cohort for five of the biomarkers. Cross-biomarker concordance analysis identified composite high (*n* = 104) and low (*n* = 110) TRAJ profiles. Key metrics for evaluating patient outcomes include Glasgow Outcome Scale Extended (GOSE), mortality, and Quality of Life after Brain Injury Overall Scale (QoLIBRI-OS) at 3, 6, and 12 months as well as a prognostic incremental value analysis using a conventional prediction model: International Mission for Prognosis and Analysis of Clinical Trials in TBI (IMPACT). High TRAJ membership is strongly associated with poor functional recovery (GOSE 1–4 at 3–12 months; odds ratio (OR) 8.79 [95% confidence interval (CI): 4.56-16.97]—12.29 [95%CI: 6.19–24.40], *P* < 0.001) and increased 180-day mortality (OR (14.84 [95%CI: 5.56–39.64], *P* < 0.001). Conversely, low TRAJ membership predicted favourable recovery (GOSE 6–8 at 3–12 months; OR 7.42 [95%CI: 3.10–17.76]—10.83 [95%CI: 3.65–32.14], *P* < 0.001) and better quality of life (QoLIBRI-OS ≥52; OR 4.98 [95%CI: 1.92–12.89], *P* < 0.01). Compared to single day-1 biomarker measurements, trajectory-based profiles yielded larger effect sizes and provided incremental prognostic value when added to the IMPACT prediction model (ΔR² 9–17%, *P* < 0.05). Overall, repeated biomarker measurements across the acute phase yield superior prognostic accuracy relative to single timepoint assessments. These findings underscore the importance of integrating longitudinal biomarker monitoring into ICU-based traumatic brain injury care and suggest that temporal trajectory profiling may improve prognostic modelling and facilitate more precise patient stratification for both clinical management and interventional studies.

## Teaser

Do *et al*. report that serum biomarker trajectory profiling on intensive care unit-admitted traumatic brain injury patients reveals distinct high and low temporal patterns associated with functional outcome and mortality. Longitudinal trajectories outperform single measurements, offering significant incremental prognostic value, supporting integration into precision traumatic brain injury care and outcome modelling.

## Graphical Abstract

**Graphical Abstract:**
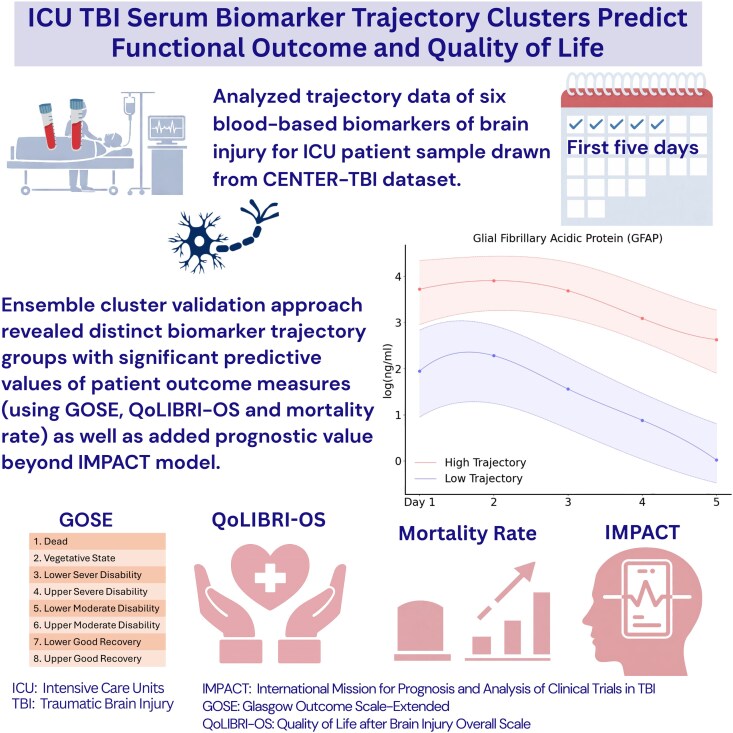

## Introduction

Traumatic brain injury (TBI) represents a major global health burden as one of the top three causes of injury-related death and disability,^1-3^ with an estimated 50–60 million new cases each year. It is also a significant economic burden, costing the global economy around US$400 billion annually. TBI is inherently heterogeneous, varying in severity, aetiology, pathology, clinical trajectory and prognosis.^4^ Although the Glasgow Coma Scale (GCS) is widely used to categorize TBI severity—mild (GCS 13–15), moderate (GCS 9–12), and severe (GCS 3–8)—it lacks the sensitivity to capture the complex and dynamic nature of brain injury.^1,3^ As a result, there is growing emphasis to go beyond the traditional severity-based approaches to seek objective, biology-driven means that complement traditional scoring and imaging to improve prognostic precision.^5^ For example, neuroimaging methods, such as CT imaging, are being used in addition to GCS scores, to determine injury severity as quantified by presence or absence of brain lesions, or the type of lesion (such as different haematoma types, subarachnoid haemorrhage, or diffuse axonal injury).^6^ CT is now considered a standard component of early TBI evaluation to detect intracranial injury and assist in determining injury severity.^7^ Blood-based biomarkers have also emerged as promising tools to complement clinical assessment, enabling more rapid efficient complimentary means to assess brain injury characteristics and monitor progression, neuroworsening, and/or secondary insults.^8^ Recently, the NIH/NINDS TBI Classification and Nomenclature Initiative has formalized a new Common Data Elements–Multidimensional Characterization (CBI-M) framework that seeks to redefine how TBI is classified.^3,9^ The four-pillar model (clinical, biomarker, imaging, and modifier domains) integrates biological and physiological data to capture the continuous spectrum of injury. The biomarker pillar of the CBI-M framework highlights validated serum proteins such as GFAP, UCH-L1, S100B, and neurofilament light chain (NfL), while the imaging pillar standardizes CT and MRI lesion descriptors derived from multicentre initiatives such as CENTER-TBI and TRACK-TBI.^10,11^ Together with demographic and physiological modifiers, this multidimensional framework enables biologically grounded phenotyping and individualized risk stratification. The current study directly aligns with this initiative by using serial serum biomarkers to define clinically meaningful subgroups of TBI patients.

Over the past two decades, extensive proteomic research has identified and characterized multiple biofluid-based protein biomarkers reflective of astrocytic, neuronal, and axonal injury.^6,12-15^ Biomarkers such as S100B and glial fibrillary acidic protein (GFAP) have been identified as astrocyte markers with diagnostic potential in TBI. S100B is detectable early post-injury and remains in clinical use, notably in the Scandinavian guidelines for initial management of minimal, mild and moderate head injuries.^16^ S100B levels, when sampled within a specific timeframe (e.g. within 3 h of injury), can help identify patients who may not need a CT scan. Despite its diagnostic promise, its interpretation is complicated by expression in peripheral tissues, particularly muscle, which can confound readings in polytrauma cases.^17^ Detectable within an hour post-injury, GFAP levels correlate with CT imaging, GCS scores, and neurosurgical interventions,^14^ showing a biphasic profile with acute and long-term elevations, useful for assessing moderate to severe TBI impact.^18^ Ubiquitin C-Terminal Hydrolase-L1 (UCH-L1) a neuron-specific enzyme constituting 1–5% of soluble brain protein, appears in circulation within one hour post-injury and scales with lesion burden.^19,20^ Neurofilament Light (NfL) is a promising biomarker for axonal injury, exhibiting a unique rise in blood levels within the first 2–3 weeks after injury.^21^ Neuron-specific enolase (NSE) is another biomarker whose blood levels are found to be elevated within the first day following a TBI.^8^ Several isoforms of the tau protein have been investigated as potential diagnostic biomarkers for neurodegenerative diseases. In the context of TBI, total tau- a microtubule-associated protein involved in axonal transport- has attracted interest as a biomarker for axonal injury. Experimental studies have shown that tau undergoes hyperphosphorylation after TBI,^22^ which promotes the formation of neurofibrillary tangles observed after moderate to severe TBI.^23^ While phosphorylated and aggregated tau species are relevant to long-term neurodegenerative changes, most clinical studies have focused on total tau. Collectively, these markers capture distinct yet complementary facets of brain injury biology and form the foundation for quantitative, mechanism-informed classification. Furthermore, the clinical utility is increasingly recognized, as demonstrated by the adoption of UCH-L1 and GFAP assays in clinical practice to assist in the diagnosis of mild TBI in patients with suspected intracranial lesions.^24,25^ However, their applicability remains limited to cases of mild TBI.

Unsupervised machine learning models can provide a robust data-driven means to learn clinically relevant subgroups that shed light on recovery dynamics across TBI severities.^26-28^ Czeiter *et al*.^8^ correlated blood biomarker data (single timepoint from less than 24 h) to TBI severity and CT imaging abnormalities. Akerlund *et al*.^27^ developed an unsupervised statistical clustering model to identify six stable TBI endotypes defined by Glasgow Coma Scale scores and metabolic stress profiles (lactate, glucose, pH, temperature). Importantly, metabolic derangement provided prognostic information independent of GCS severity, demonstrating the value of incorporating metabolic factors into TBI classification. In their exploratory work, Yeboah *et al*.^28^ applied an ensemble clustering model to identify TBI subgroups based on baseline data like injury assessment information, metabolic, liver, haematologic functions and CT scan results.^28^ Some studies have analysed repeated serum biomarker measurements to identify TBI subgroups with similar patterns using group-based multi-trajectory (TRAJ) modelling.^21,29-33^ Wang *et al*.^21^ conducted a comprehensive study to characterize the temporal profiles of NfL and phospho-neurofilament-heavy (pNFH) in both CSF and serum, assessing their diagnostic and prognostic potential in moderate-to-severe TBI. The study measured serum biomarkers across acute (daily), subacute (weekly), and chronic (monthly) time frames, correlating them to functional outcome. Results demonstrated that serum NfL levels, and to a lesser extent pNFH levels, peaked during the subacute phase and were robustly associated with poor outcomes at 6 and 12 months.^21^

This study builds upon the current state of the art by conducting cluster analysis (unsupervised machine learning) on serum biomarker trajectory data from the first 5 days of ICU patients treated at Level 1 trauma centres. To anchor the biomarker analyses to structural injury, we focus on patients with CT-positive (CT+) TBI (i.e. those with radiologically confirmed intracranial abnormalities) admitted to the ICU. These patients tend to exhibit higher biomarker levels and poorer long-term outcomes than CT-negative cases.^34,35^ Restricting the cohort to CT+ enhances interpretability and aligns with the CBI-M imaging pillar, which employs standardized lesion descriptors as the structural reference standard. The overall aim of this study is to identify clinically meaningful subgroups that may help predict long-term outcomes. We also examine the clinical utility of repeated measurements of blood-based TBI biomarkers to predict patient outcomes, compared to relying on a single measurement taken during the early acute phase (e.g. ≤ 1 day). We hypothesize that membership in these subgroups may predict and/or reflect differences in patient outcomes. The biomarker trajectories are analysed to explore their correlation with TBI subphenotypes, serving as potential predictors of patient outcomes. Key metrics for evaluating patient outcomes include Glasgow Outcome Scale Extended (GOSE),^36,37^ mortality,^38^ and Quality of Life after Brain Injury Overall Scale (QoLIBRI-OS)^39,40^ at 3, 6, and 12 months, as well as a prognostic incremental value analysis using an International Mission for Prognosis and Analysis of Clinical Trials in TBI (IMPACT)^41^ model. The novelty of this study lies in its hypothesis that integrating the composite temporal trajectories of TBI biomarkers across ICU patients provides superior insight compared to single timepoint (day-1) biomarker measurement. By capturing the dynamics of biomarker profiles, this approach enables identification of patients who sustain persistently elevated biomarker levels—patients who are more likely to experience delayed mortality, poorer GOSE scores, and reduced quality of life. Through longitudinal modelling and characterization of biomarker trajectories, this work advances the paradigm of multidimensional, biology-driven prognostication envisioned by NINDS, establishing an analytical framework for precision classification that augments established existing clinical and imaging models such as IMPACT.^42,43^

## Materials and methods

### Subject and data inclusion

**Figure 1:**
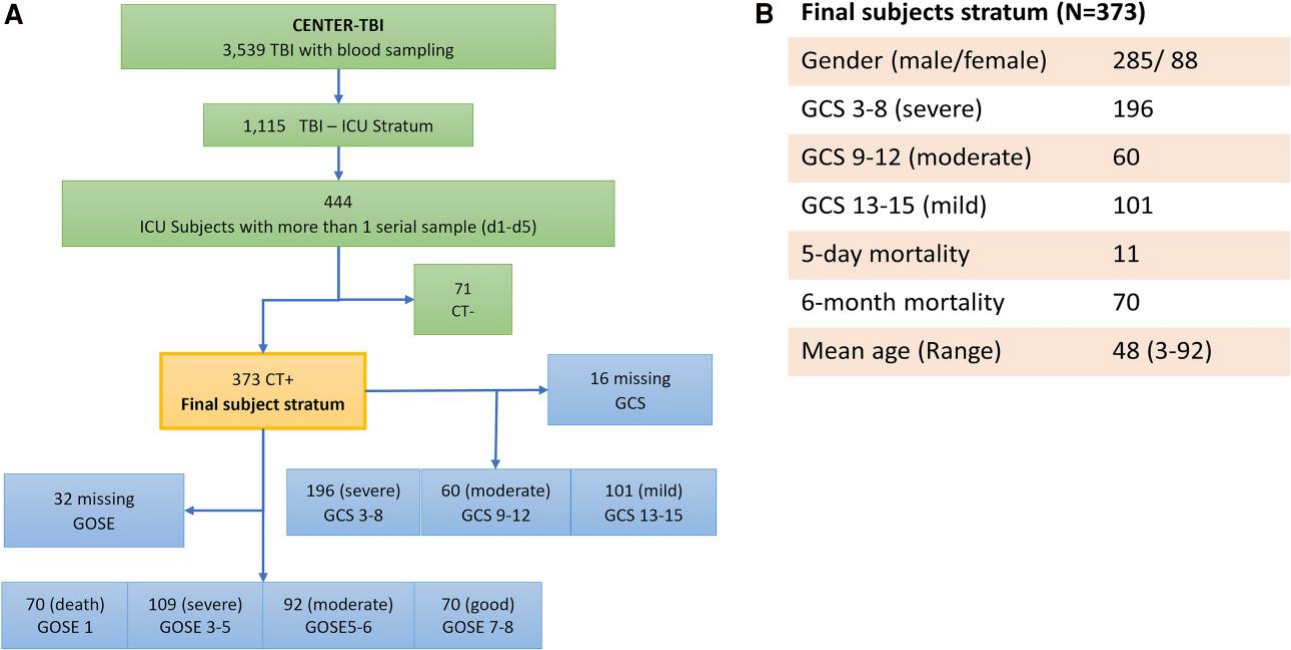
**Study selection and demographic characteristics. (A)** CONSORT diagram for study subject selection from CENTER-TBI. **(B)** Demographic and injury characteristics of the final subject stratum.

The study sample is drawn from the Collaborative European NeuroTrauma Effectiveness Research (CENTER-TBI) Study (**NCT02210221,** year 2014–2021),^8,44^ a prospective observational clinical and biomarker study of patients with TBI, conducted in 65 clinical sites from 17 European countries and Israel. All associated data used in this study were obtained from Biobank Antwerp, Antwerp, Belgium ID: BE 71030031000.^45^ Details of the protocol and clinical data have been previously published.^8,44^ Patients with all severities of TBI presenting to a study centre within 24 h of injury and scheduled for CT scanning were enrolled, stratified by care path (emergency department, hospital admission and intensive care unit (ICU)). The only exclusion criterion was a severe pre-existing neurological disorder. This study focuses on the ICU subset (i.e. patients with TBI receiving treatment in the ICU at Level 1 trauma centres). Of the ICU stratum (*N* = 1115 with biomarkers sample collected), 444 subjects had at least two-timed serum samples within 5 days post-injury—Days 1 (up to 24 h), 2 (25–48 h), 3 (49–72 h), 4 (73–96 h), and 5 (97–120 h). Thus, the minimal number of data points are two: one acute (day 1) data point and at least one post-acute data point within Days 2–5. Since the study sample was drawn from the ICU stratum, the premise is that, although these TBI patients all have significant brain injuries, there may still be latent subclasses distinguishable based on their first 5-day biomarker trajectories. To further stress-test this hypothesis, we excluded the 71 ICU subjects who were cranial CT negative, as some of these patients more likely had milder injuries (Fig. 1A).

We analysed the d1-d5 trajectories of six biomarkers (S100B, NSE, GFAP, NfL, Tau, and UCH-L1) of the remaining 373 ICU-admitted TBI patients with traumatic intracranial findings on CT (CT+) (see Fig. 1A). Demographic and baseline characteristics of the 373 CT + subjects are presented in Fig. 1B. 372 of the study sample are of white European ethnicity. The control group for establishing a normative baseline consisted of serum samples (commercial source—BioIVT) from 122 healthy subjects for biomarkers GFAP, NfL, Tau, and UCH-L1 (median age 45.3; male 62.10% *P* < 0.01), and 60 subjects for NSE and S100B (median age 48.7; male 66.67%).

### Biomarker assaying

UCH-L1, GFAP, NfL and Tau biomarkers were assayed using the Quanterix SIMOA N4PB Neurology 4-plex assay (Catalog # 103345) and the Quanterix SR-X analyser at the University of Florida’s laboratory of Program for Neurotrauma, Neuroproteomics & Biomarker Research and Department of Neurobiology, Moorehouse School of Medicine. S100B and NSE were assayed using the Roche Elecsys platform at University of Pecs.^8^ The detailed methods and analytic performance of each assay have been described previously.^8,44^ Blood samples were collected into serum separator tubes (red-top, no anticoagulant). Samples were allowed to clot at room temperature and were then centrifuged at 3500 × g for 5 min. The resulting supernatant was carefully transferred to sterile cryovials and designated as serum. The average interval from venipuncture to initial freezing at −80°C was 1 h and 10 min (mean ± SD: 1.10 ± 30 min) to minimize pre-analytical variability. Serum GFAP, UCH-L1, NfL, and total Tau concentrations were measured using the Quanterix Simoa™ platform according to the manufacturer’s protocol. Samples were analysed at a 1:4 dilution, corresponding to 12.5 μL of serum per 50 μL final assay incubation volume. Serum S100B and NSE levels were quantified on the Roche COBAS® automated immunoassay system, following the manufacturer’s standardized procedures. Required sample input volumes were 30 μL for S100B and 50 μL for NSE, respectively. Analytical reproducibility was confirmed through technical replicate testing (≈20% of samples, CV < 15%), demonstrating stable assay performance. The analysts who performed the biomarker assays on the serum samples were blinded to the clinical data of the subjects.

### Data curation

Not all patients had a complete 5-day profile for all biomarkers (see Supplementary Table 1). Missing biomarker values (15% across the entire dataset) were imputed following a Missing at Random (MAR) pattern assumption using the iterative imputer method based on *k*-nearest neighbour (KNN).^46,47^ Each missing time point is imputed using values from nearest neighbours (based on Euclidean distance) that have a value for that day. Demographic and injury information were included along with the biomarker data, to ensure a more accurate imputed estimate.

### Cluster analysis

Clustering analysts, who performed the ML trajectory groupings, were blinded to the clinical data of the subjects. We applied an ensemble clustering and validation model^48^ to determine the optimal trajectory subgroups across the five daily time points using five commonly used clustering algorithms: *k*-means algorithm,^49^ Spectral,^50^ Agglomerate,^49^ Gaussian mixture,^51^ and Sequential *k*-means clustering (using the Kullback–Leibler distance rather than the Euclidean distance^52^). For each algorithm, the number of clusters (*k*) is set a priori and varied *k* from 2 to 5, given the relatively small number of samples. Cluster analysis yields different sets of clusters (homogeneous subgroups) by varying a given parameter for the same algorithm. To determine the optimal clustering outcome, we apply an ensemble cluster validation model based on internal validation metrics to select the most optimal result.^48^ These metrics assess the optimal clustering solution by evaluating different aspects of cluster compactness and separation.^53^ It leverages the strengths of seven internal validation metrics (Silhouette, Davies-Bouldin, Xie-Beni, Calinski-Harabasz, Cluster validation index based on nearest neighbours, Mean index accuracy, and Similarity matrix index)^53-59^ to determine the most optimal result using an aggregated ranking approach, as described in Supplementary Table 2. To derive the composite clusters across the set of biomarker subgroups, we defined the composite subgroup as an intersection of all the patients who were in the same cluster for each biomarker.

### Outcome measures

Three outcome measures (Glasgow Outcome Scale Extended (GOSE),^36,37^ Quality of Life after Brain Injury Overall Scale (QoLIBRI-OS),^39,40^ and mortality) were used to assess clinical relevance of the cluster analysis model. GOSE is a global outcome assessment of a TBI patient based on an 8-point scale: 1- dead, 2-vegetative state, 3- lower severe disability, 4- upper severe disability, 5- lower moderate disability, 6-upper moderate disability, 7-lower good recovery, and 8- upper good recovery. It is recognized as the gold standard for TBI global outcome by the FDA.^36,37^ (Note that for the CENTER-TBI study, the categories vegetative state and lower severe disability were combined as these could not be differentiated on assessments performed by postal questionnaire.) While GOSE captures global and functional outcomes, long-term recovery requires considering the quality of life of the patients. Stocchetti and Zanier^60^ highlighted the chronic, long-term impacts of TBI, including physical, cognitive, and psychological challenges, and the limitations of traditional scales in tracking recovery.

To date, there are few studies examining if post-TBI blood-based biomarker patterns can predict quality of life outcome. Hence, we employed the QoLIBRI-OS, a six-item measure evaluating satisfaction across key domains relevant to individuals with TBI—physical health, cognition, emotions, daily functioning, personal and social life, and future outlook—using a 5-point scale ranging from ‘not at all satisfied’ to ‘very satisfied’.^60,61^ Item scores are summed and transformed into a total score on a 0–100 scale, with higher scores indicating better quality of life. The GOSE and QOLIBRI-OS scores were collected at 3-, 6-, and 12-months post-injury. To assess mortality, we examined two scenarios: early mortality, defined as deaths occurring within the first 5 days post-injury, and overall mortality, focusing on deaths occurring within the first 180 days post-injury, as most fatalities occur within this timeframe. Note that some of the selected subjects belonged to the early death category (died within 5 days). These patients were not excluded from the trajectory class modelling, thus enabling us to evaluate whether any trajectory class membership was associated with early mortality. To verify the clinical relevance of the resulting trajectory classification, we also evaluated its incremental predictive power beyond a conventional prediction model, IMPACT. The IMPACT score was developed by Steyerberg *et al*.^41^ as part of a large collaborative effort to improve outcome prediction in TBI patients with admission GCS ≤ 12.

### Statistical analysis

We applied the Mann–Whitney *U* test to compare group medians, given the non-normal distribution of the data, to evaluate significant differences in biomarker trajectories between clusters.^26^ Tests for a significant association between the cluster group and binarized GOSE outcomes were conducted using a $\chi^{2}$ test for independence for each biomarker and time point. A favourable global outcome indicative of ***Good Recovery*** is defined as a GOSE score of 7–8 (versus 1–6), and unfavourable outcome indicative of ***Poor Recovery*** as a GOSE score of 1–4 (versus 5–8). Note that for analyses of good recovery (GOSE scores 7–8 versus 1–6), the OR was calculated with the low TRAJ class as the reference. An OR significantly greater than 1 indicates that the low TRAJ class has higher odds of achieving a good recovery outcome compared to the high TRAJ class. Likewise, when testing for poor recovery outcome (GOSE scores 1–4 or not i.e. scores 5–8), the OR is calculated with the high TRAJ class as the reference.

To examine whether using the biomarker TRAJ clusters based on repeated measurements is superior to using the biomarker level at a single acute timepoint (Day 1) in predicting outcome, we also conducted a similar cluster analysis based on Day 1 biomarker levels for each of the six biomarkers. The odds ratio was calculated with a 95% confidence interval and Fisher’s exact *P*-value to assess whether it differed significantly from Day-1. The Breslow-Day test for homogeneity of odds ratios was used to compare differences in odds ratios between clustering models based on Day-1 and 5-day (Days 1–5) biomarker data.

To assess the incremental value of the TRAJ membership on the IMPACT prediction model, individual IMPACT scores for both the Core and Extended models were calculated. The GCS motor score in the IMPACT model was replaced with the GCS overall score, as the patient sample is broader than that of moderate to severe TBI. Predictions for 6-month mortality and 6-month unfavourable GOSE outcome were generated using logistic regression models to compare predictive performance and assess the incremental value of the biomarker-derived TRAJ clusters. First, a base model was fitted using each IMPACT score (Core or Extended) as the sole predictor. Next, a combined model was generated that included the IMPACT score and the cluster variable as predictors. Nagelkerke’s *R*^2^ was calculated as a goodness-of-fit metric (ranging between 0 and 1, with values closer to 1 indicating a better fit). The difference (Delta or Δ*R*^2^) in Nagelkerke’s *R*^2^ when the cluster variable is added to the IMPACT model indicates the incremental improvement in model fit. An approximate 95% CI of Delta *R*^2^ generated via bootstrap.^62^ The area under the receiver operating characteristic curve (AUC) is also calculated and the difference in AUC (Delta or Δ AUC) when the cluster variable is added to the IMPACT model to examine differences in discriminative performance. Differences in AUC tested with the DeLong method.^63^ Note this study followed the TRIPOD reporting guidelines.^64^

## Results

The 373 CT + subjects cohort showed an imbalanced distribution of GCS categories (3–8 (*n* = 196), 9–12 (*n* = 60), and 13–15 (*n* = 101)) (see Fig. 1B). Nevertheless, subjects are almost evenly distributed for GOSE 1–4 (*n* = 179) and GOSE 5–8 (*n* = 162). In contrast, much fewer subjects are in the GOSE 7–8 category (*n* = 70) versus GOSE 1–6 (*n* = 271).

### Cluster analysis of biomarker trajectory within the first 5 days

**Figure 2:**
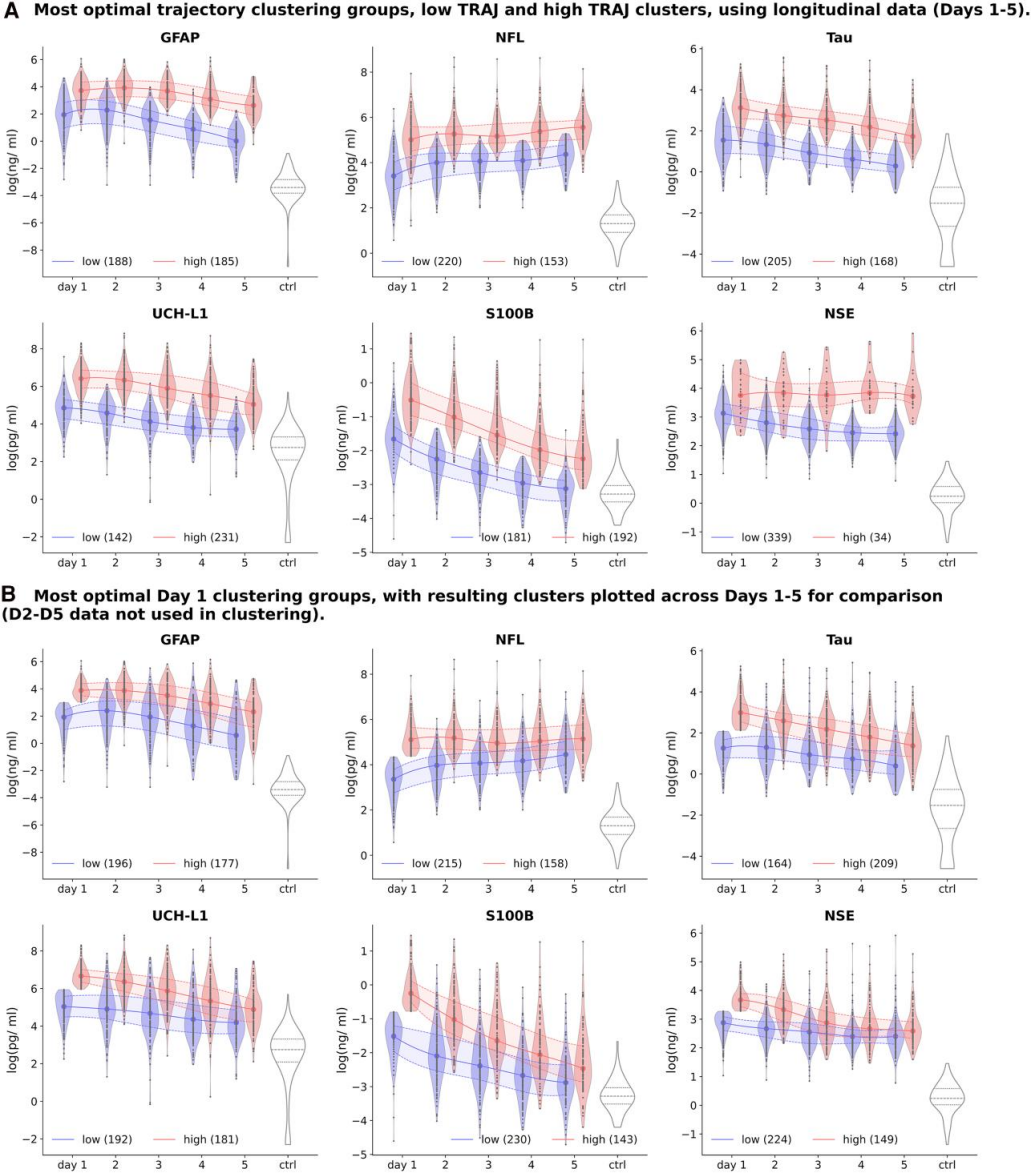
**Visualization of cluster analysis outcome across the 6 TBI biomarkers.** (**A**) Most optimal trajectory clustering groups: low TRAJ and high TRAJ clusters using longitudinal data (Days 1–5). **(B)** Most optimal Day 1 clustering groups, with resulting clusters plotted across Days 1–5 for comparison (D2-D5 data not included in clustering analysis). Violin plots show median, minimum and maximum values, with frequency indicated by width. Each datapoint represents the biomarker level value of an independent patient sample by day. Sample sizes of class memberships are indicated by the numbers in brackets. Note that there were significant differences in the median biomarker levels between high and low TRAJ groups for each biomarker across all time points based on the Mann–Whitney *U*-test (*P* < 0.001).

Applying the five varied clustering algorithms to derive biomarker trajectory models for identifying two or more homogeneous subgroups of severe TBI patients yielded 20 distinct model variants per biomarker, each comprising between 2 and 5 subgroups (classes). Models were ranked based on their performance within the ensemble validation framework, and a composite sum score was computed for each. The clustering model with the highest sum score was identified and selected for further analysis per biomarker (Supplementary Table 3). Figure 2A illustrates the distribution of the most optimal trajectory clustering groups (TRAJ) for all 6 TBI biomarkers. GFAP, NfL, and NSE are best-fitted to the Agglomerative 2-classes model, S100B and UCH-L1 to *k-*means 2-class model, while Tau is best-fitted to Spectral 2-class model. Supplementary Fig. 1 shows the biomarker temporal data distribution of all six biomarkers and their levels with respect to those of healthy controls to provide a reference baseline for biomarker level and to illustrate the magnitude of differences between healthy individuals and TBI subjects as also evident from Fig. 2A.

We observe that the optimal 2-class clustering result split the patients into two distinct trajectories, a low TRAJ class and a high TRAJ subclass per biomarker (Fig. 2A). For GFAP, NfL, Tau, UCH-L1, and S100B, each of the subclasses represents at least 38% of all 373 patients. The only exception is NSE, with its high TRAJ C2 class having only 34 patients (9.1%). The Mann–Whitney *U* tests revealed significant differences in median biomarker levels between high and low clusters across Days 1–5 (Table 1).

**Table 1:** Statistical analysis of trajectory biomarker clusters using Mann–Whitney non-parametric test

	GFAP				NFL			
	C1 Low TRAJ (*N* = 188)		C2 High TRAJ (*N* = 185)		C1 Low TRAJ (*N* = 220)		C2 High TRAJ (*N* = 153)	
Day	Median	95% CI	Median	95% CI	Median	95% CI	Median	95% CI
**1**	7.02	6.18–8.47	41.32 †††	36.63–48.69	30.29	26.95–34.03	149.42†††	123.97–183.87
**2**	9.79	7.23–12.17	49.88 †††	42.43–62.7	54.72	46.48–61.7	193.33†††	171.34–213.86
**3**	4.75	3.79–6.51	40.02 †††	32.15–50.22	58.03	50.81–66.28	175.23†††	151.04–199.05
**4**	2.42	1.66–3.02	21.94 †††	18.78–27.28	59.97	54.66–66.65	215.24†††	184.71–248.59
**5**	1.02	0.84–1.43	13.87 †††	11.11–15.68	78.75	68.46–85.2	258.94 †††	222.67–280.74

	Tau				UCH-L1			
	C1 low TRAJ (*N* = 205)		C2 high TRAJ (*n* = 168)		C1 low TRAJ (*N* = 142)		C2 high TRAJ (*N* = 231)	
**1**	4.7	4.04–5.34	22.73 †††	19.31–24.94	128.05	107.65–151.17	610.92 †††	567.03–719.74
**2**	3.79	3.16–4.37	15.57 †††	14.44–17.08	98.08	86.61–121.69	562.22 †††	485.09–626.22
**3**	2.54	2.24–2.8	12.49 †††	11.32–13.82	62.13	53.51–76.92	362.8 †††	317.02–455.08
**4**	1.86	1.68–2.17	8.87 †††	7.8–10.09	44.54	38.74–56.61	247.45 †††	196.27–285.42
**5**	1.34	1.25–1.63	5.62 †††	4.8–6.52	41.25	36.47–46.53	155.7 †††	123.32–173.82

	S100B				NSE			
	C1 low TRAJ (*N* = 181)		C2 high TRAJ (*N* = 192)		C1 low TRAJ (*N* = 339)		C2 high TRAJ (*N* = 34)	
**1**	0.19	0.17–0.21	0.6 †††	0.51–0.67	22.91	21.36–24.46	42.68 †††	30.0–73.28
**2**	0.1	0.1–0.12	0.36 †††	0.32–0.41	16.54	15.87–17.61	46.68 †††	36.12–58.26
**3**	0.07	0.06–0.08	0.21 †††	0.19–0.27	13.26	12.39–14.14	43.16 †††	34.45–55.64
**4**	0.05	0.05–0.06	0.14 †††	0.13–0.17	11.65	10.98–12.26	46.78 †††	38.48–64.91
**5**	0.04	0.04–0.05	0.11 †††	0.1–0.12	11.23	10.72–11.82	41.36 †††	33.54–51.15

Median levels of biomarkers and CI are shown (GFAP, S100B, NSE ng/mL, NFL, Tau, UCH-L1 pg/mL).

††† *P* < 0.001 statistically significant between high and low clusters.

### Outcome prediction based on biomarker TRAJ clustering

#### Global outcome by Glasgow outcome scale extended (GOSE)

We analysed the low and high TRAJ classes for each biomarker in terms of global patient outcome using GOSE scores present at 3-, 6-, and 12 months post-injury based on *χ*² tests of independence and odds ratios (OR) with 95% confidence intervals (CIs) (see Fig. 1 and Table 2). For GFAP, UCH-L1, NfL, Tau, and S100B, the low TRAJ class shows significantly higher odds of a good recovery global outcome (GOSE 7–8) at 3, 6, and 12 months (Table 2). The exception is the NSE biomarker, in which the low TRAJ was not significantly associated with good recovery outcome at 3 and 6 months (*χ*2 test and OR). All biomarkers’ high TRAJ class showed significantly higher odds of a poor recovery global outcome (GOSE 1–4) at 3, 6 and 12 mo. NSE also had a relatively small high TRAJ class size (*n* = 34) compared to the low TRAJ class counterpart (*n* = 339).

**Table 2:** Statistical analysis of patient outcome by trajectory (D1–D5) clusters as qualified by good recovery (GOSE 7–8 versus GOSE 1–6 counterparts) and poor recovery (GOSE 1–4 versus GOSE 5–8 counterparts)

Timepoint (month)		Low TRAJ/Good GOSE (7–8—True)		High TRAJ/Poor GOSE (1–4—True)	
		OR (95%CI)	χ^2^	OR (95%CI)	χ^2^
GFAP clusters					
	3	5.92 (2.67–13.1) †††	21.64 †††	3.75 (2.3–6.12) †††	28.3 †††
	6	3.75 (2.08–6.74) †††	19.82 †††	3.58 (2.29–5.6) †††	31.16 †††
	12	3.11 (1.88–5.15) †††	19.2 †††	4.58 (2.9–7.24) †††	43.22 †††
NfL clusters					
	3	5.87 (2.42–14.25) †††	17.27 †††	5.69 (3.26–9.96) †††	40.23 †††
	6	4.52 (2.32–8.8) †††	20.97 †††	4.63 (2.89–7.41) †††	41.84 †††
	12	4.82 (2.69–8.61) †††	29.9 †††	5.01 (3.15–7.98) †††	47.27 †††
Tau clusters					
	3	4.61 (2.08–10.21) †††	15.12 †††	3.49 (2.11–5.76) †††	23.95 †††
	6	3.14 (1.73–5.7) †††	14.06 †††	3.75 (2.38–5.9) †††	32.6 †††
	12	3.26 (1.93–5.52) †††	19.38 †††	4.46 (2.83–7.05) †††	41.8 †††
UCH-L1 clusters					
	3	5.45 (2.75–10.82) †††	25.7 †††	3.56 (2.21–5.72) †††	27.38 †††
	6	2.99 (1.74–5.13) †††	15.51 †††	3.13 (1.98–4.94) †††	23.6 †††
	12	3.06 (1.88–4.99) †††	19.81 †††	4.92 (3.01–8.05) †††	41.74 †††
S100B clusters					
	3	4.82 (2.31–10.05) †††	18.76 †††	4.28 (2.62–6.99) †††	34.35 †††
	6	2.96 (1.69–5.18) †††	14.2 †††	3.84 (2.45–6.02) †††	34.52 †††
	12	2.87 (1.75–4.72) †††	17.08 †††	4.32 (2.73–6.82) †††	39.82 †††
NSE clusters					
	3	2.69 (0.62–11.65)	1.24	3.99 (1.37–11.66) †	6.33 †
	6	4.39 (1.02–18.82)	3.76	3.12 (1.37–7.14) ††	6.93 ††
	12	14.06 (1.89–104.39) ††	9.88 ††	3.46 (1.56–7.69) ††	9.09 ††
CB^a^ clusters					
	3	10.83 (3.65–32.14) †††	23.3 †††	10.49 (4.96–22.18) †††	42.9 †††
	6	7.42 (3.10–17.76) †††	22.75 †††	8.79 (4.56–16.97) †††	44.56 †††
	12	7.99 (3.71–17.20) †††	31.28 †††	12.29 (6.19–24.40) †††	56.55 †††

^a^CB: composite based.

† *P* < 0.05, †† *P* < 0.01, ††† *P* < 0.001.

As NSE did not show fully robust GOSE prediction performance, we subsequently excluded it from the remaining analysis. There was significant overlap between the low and high trajectory groups of the other five biomarkers. One hundred and ten subjects belong to all 5 low-trajectory groups, while 104 subjects belong to all 5 high-trajectory groups (see Supplementary Fig. 3). The remaining mixed group has 159 subjects. We assigned the all-low TRAJ and all-high TRAJ as ‘Composite Biomarker’ (CB) low TRAJ and high TRAJ classes, respectively (Table 2). The CB high TRAJ class showed the most robust prediction of poor recovery outcome with odds ratio of 8.8 (4.56–16.97) to 12.3 (6.19–24.40) for GOSE 1–4). Similarly, CB low TRAJ class showed the most robust prediction of *good recovery* outcome with odds ratio of 8.0 (3.71–17.20)-10.8 (3.65–32.14) for GOSE 7–8).

#### Prediction of mortality at 6 months using biomarker trajectory

**Figure 3:**
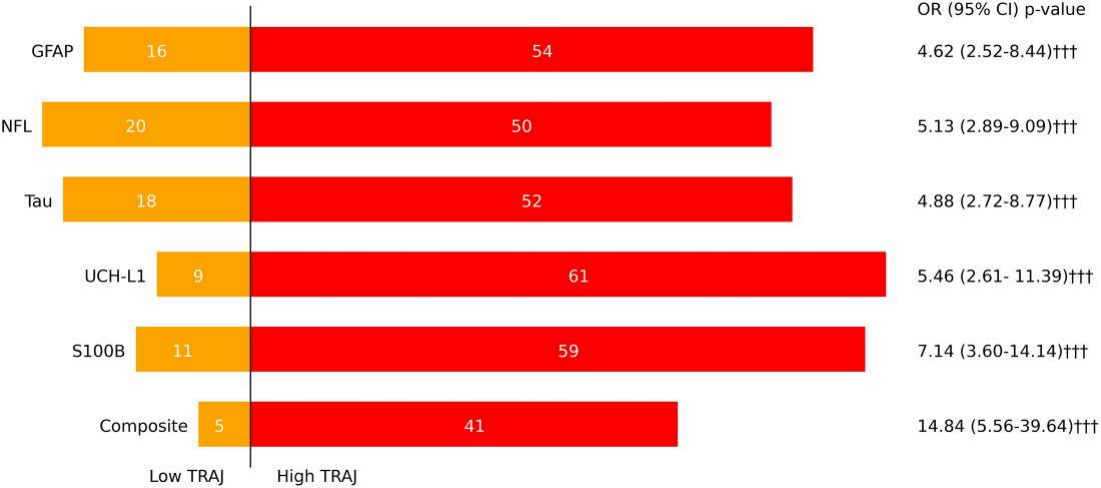
**Mortality counts at 6 months for each trajectory cluster and odds ratio (OR) of mortality in the high trajectory group.** (††† *P* < 0.001).

For 6 month mortality prediction (Fig. 3), each of the five biomarkers exhibited higher odds of mortality in the high TRAJ group, with strongly significant OR (4.62 (2.52–8.44) to 7.14 (3.60–14.14), *P* < 0.001). The composite biomarker group exhibited an even stronger OR (14.84 (5.56–39.64), *P* < 0.001). Figure 3 further shows 6 months mortality subcategory distribution in low- and high-trajectory groups for each biomarker.

#### Single acute timepoint (D1) clustering versus 5-day trajectory profile clustering

Figure 2B illustrates the clusters obtained based on a single acute timepoint (D1). Each subclass’s respective biomarker levels are shown across Days 1–5 though only D1 data was utilized for clustering. (An alternative visualization of the clustering results can be found in Supplementary Fig. 4 for 5-day clustering and Supplementary Fig. 5 for D1 clusters.) We observe that there are significant differences in the median biomarker levels between high and low clusters for each biomarker across all time points (Mann–Whitney *U*-test).

From these figures, we observe more overlap in the interquartile ranges for the D1 clusters, compared to the 5-day clusters. Table 3 shows the performance of D1 low and high clustering subgroups in predicting good recovery outcome (GOSE 7–8) and poor recovery outcome (GOSE 1–4), respectively. A significant association between the trajectory class and GOSE outcome was found for GFAP, NfL, Tau, and UCH-L1 at all three time points (*χ*^2^ test and OR). When predicting good GOSE (scores 7–8) from the low TRAJ class, OR scores were in the range of 1.69 (1.04–2.75) to 4.28 (2.16–8.46) for these four biomarkers, while OR scores were in the range of 2.03 (1.31–3.14) to 3.03 (1.9–4.84) for these biomarkers when predicting poor recovery (GOSE scores 1–4) from the high TRAJ class. The odds ratios based on D1 clustering were consistently lower than their respective 5-day low TRAJ subgroup in predicting good recovery GOSE (OR: 3.0 (1.74–5.13) to 5.9 (2.67–13.1)) and high TRAJ subgroup for predicting poor GOSE (OR: 3.1 (1.98–4.94) to 5.7 (3.26–9.96)) for these four biomarkers (Table 2). The S100B clustering results for predicting good recovery GOSE from the low TRAJ class were not significant for the D1 clustering, but they were significant for the 5-day clustering (*χ*^2^, odds ratio). Both clustering results exhibited significance for S100B predicting poor recovery GOSE from the high TRAJ class, and neither showed consistent importance for NSE.

**Table 3:** D1 (acute single timepoint) clusters statistical analysis of patient outcome as qualified by good recovery outcome (GOSE 7–8 versus GOSE 1–6 counterparts) and poor recovery outcome (GOSE 1–4 versus GOSE 5–8 counterparts)

Timepoint (month)		Low TRAJ/good GOSE (7–8—True)		High TRAJ/poor GOSE (1–4—True)	
		OR (95%CI)	χ^2^	OR (95%CI)	χ^2^
GFAP clusters					
	3	3.41 (1.67– 6.96)†††	11.31 †††	2.87 (1.77–4.64)†††	18.06 †††
	6	2.3 (1.31–4.01)††	8.03 ††	2.55 (1.65–3.96)†††	17.01 †††
	12	1.69 (1.04–2.75)†	4.09 †	2.54 (1.64–3.94)†††	16.91 †††
NfL clusters					
	3	2.81 (1.38–5.74)††	7.67 ††	2.37 (1.46–3.85)†††	11.77 †††
	6	2.17 (1.23–3.84)††	6.59 †	2.46 (1.58–3.83)†††	15.32 †††
	12	2.36 (1.42–3.93)†††	10.34 ††	2.45 (1.58–3.8)†††	15.35 †††
Tau clusters					
	3	4.28 (2.16–8.46)†††	18.19 †††	3.03 (1.9–4.84)†††	21.08 †††
	6	2.6 (1.51–4.46)†††	11.53 †††	2.03 (1.31–3.14)††	9.6 ††
	12	2.49 (1.53–4.04)†††	13.1 †††	2.26 (1.46–3.52)†††	12.56 †††
UCH-L1 clusters					
	3	3.65 (1.79–7.46)†††	12.8 †††	2.79 (1.73–4.49)†††	17.35 †††
	6	2.51 (1.44–4.39)††	9.98 ††	2.79 (1.8–4.33)†††	20.43 †††
	12	2.55 (1.55–4.2)†††	13.18 †††	2.57 (1.66–3.98)†††	17.26 †††
S100B clusters					
	3	1.55 (0.79–3.02)	1.28	2.29 (1.39–3.77)††	10.12 ††
	6	1.37 (0.79–2.39)	0.98	1.8 (1.15–2.8)†	6.23 †
	12	1.44 (0.87–2.37)	1.71	2.1 (1.35–3.27)††	10.22 ††
NSE clusters					
	3	2.65 (1.27–5.53)††	6.28 †	2.23 (1.36–3.65)††	9.57 ††
	6	1.76 (0.99–3.12)	3.3	1.33 (0.86–2.06)	1.35
	12	1.27 (0.77–2.07)	0.66	1.55 (1.0–2.41)	3.46
CB^a^ clusters					
	3	4.13 (2.06–8.29) †††	16.52 †††	3.08 (1.92–4.94) †††	21.51 †††
	6	2.4 (1.4–4.13) ††	9.54 ††	2.48 (1.6–3.84) †††	16.02 †††
	12	2.1 (1.3–3.39) ††	8.52 ††	2.68 (1.72–4.18) †††	18.59 †††

† *P* < 0.05, †† *P* < 0.01, ††† *P* < 0.001.

^a^CB: composite based.

D1 composite biomarkers low TRAJ and high TRAJ have OR in the range of 2.1 (1.3–3.39) to 4.1 (2.06–8.29) and 2.5 (1.6–3.84) to 3.1 (1.92–4.94) for good and poor outcomes at 3 to 12 mo., respectively (Table 3). The odds ratios based on D1 composite biomarkers were lower than their respective 5-day low TRAJ subgroup and high TRAJ subgroup composite biomarkers (with OR of 8.0 (3.71–17.20) to 10.8 (3.65–32.14) and 8.8 (4.56–16.97) to 12.3 (6.19–24.40) for good and poor outcome, respectively) (Table 2). Testing for statistically significant differences in the odds ratios between the D1 and 5-day clusters was conducted via the Breslow-Day test (Supplementary Table 4). Although results were mixed for individual biomarkers, the odds ratios for the composite biomarkers were significantly different between the D1 and 5-day clusters, apart from the 3-months’ time point, for the comparison of predicting good recovery GOSE from low TRAJ class. When predicting poor recovery GOSE from the high TRAJ class, the odds ratios for the composite biomarkers were significantly different between the D1 and 5-day clusters for all three time points. The OR was always higher in the 5-day clusters compared to D1 clusters in the significant comparisons (Supplementary Table 4).

#### Quality of life (QoL) in brain injury

The QoL measure, as quantified by QoLIBRI-OS, was dichotomized with ≥ 52 as cut-off for better QoL and < 52 as poorer QoL.^34,65,66^ Table 4 shows the odds ratio and *χ*^2^ statistic for testing for the association between TRAJ class membership and QoL outcome at 3-, 6-, and 12 months post-injury. The OR is calculated for predicting the poorer QoL class with the high TRAJ class as the reference. We observe that for five biomarkers (GFAP, NfL, Tau, UCH-L1, S100B), the high TRAJ class has significantly higher odds of poorer QoL compared to the low TRAJ class (OR 2.35 (1.22–4.51) to 2.93 (1.51–5.66), *P* ≤ 0.05–0.01) at 3 months post-injury. The composite biomarker high TRAJ class has the strongest OR (4.98 (1.92–12.89), *P* < 0.01) for predicting poorer QoL at 3 months post-injury. No significant associations were found at 6- and 12 months post injury for the NSE biomarker at any of the three time points.

**Table 4:** Odds ratio and χ2 test for predicting poor QoLIBRI-OS (1–51) at 3, 6, and 12-months post-injury based on high biomarker TRAJ cluster membership. Missing data not included in analysis

Biomarker	3-month		6-month		12-month	
	OR (95% CI)	χ^2^	OR (95% CI)	χ^2^	OR (95% CI)	χ^2^
CB^a^	4.98 (1.92–12.89) ††	10.21††	2.22 (0.88–5.64)	2.12	2.29 (0.88–5.99)	2.13
GFAP	2.85 (1.47–5.54) ††	8.85††	1.39 (0.69–2.81)	0.55	1.80 (0.90–3.60)	2.21
NfL	2.57 (1.27–5.17) †	6.20†	1.89 (0.92–3.90)	2.4	1.75 (0.85–3.57)	1.83
Tau	2.37 (1.22–4.59) †	5.78†	1.50 (0.74–3.03)	0.9	1.73 (0.86–3.47)	1.85
UCH-L1	2.35 (1.22–4.51) †	5.88†	1.58 (0.79–3.17)	1.24	2.19 (1.09–4.38) †	4.26†
S100B	2.93 (1.51–5.66) ††	9.43††	1.37 (0.69–2.72)	0.51	1.20 (0.60–2.37)	0.11
NSE	1.93 (0.50–7.47)	0.38	1.35 (0.32–5.62)	0	2.98 (0.76–11.63)	1.61

QoLIBRI-OS data were available for 159, 164, and 158 out of 373 total subjects at 3, 6, and 12 months, respectively.

^a^CB: composite based.

† *P* < 0.05, †† *P* < 0.01, ††† *P* < 0.001.

#### Assessing the incremental value of 5-day biomarker trajectory clustering on the IMPACT model

**Figure 4:**
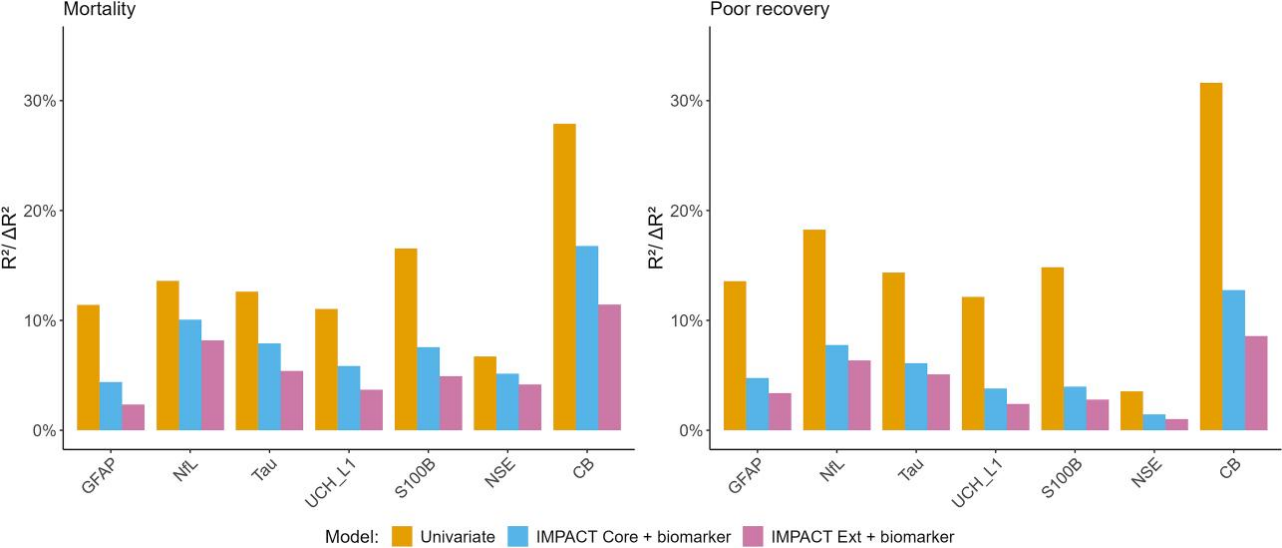
**Incremental value analysis of TRAJ clustering over IMPACT prediction model (core and extended variants) for both GOSE (poor recovery) and mortality outcome.** The first bar shows the univariate Nagelkerke *R*^2^ of the biomarker, while the other two bars represent Δ*R*^2^ values of the biomarker, representing the additional variance explained in combination with the IMPACT core and extended models. For each individual biomarker, *N* = 373 while *N* = 214 for the composite biomarker group.

The incremental value analysis evaluates the added value of the TRAJ clusters over the IMPACT core and extended models for both poor recovery (GOSE) and mortality outcomes (Fig. 4 and Supplementary Table 5). Differences in Nagelkerke’s *R*^2^ and AUC quantify the value added by the TRAJ clusters. Biomarker trajectories demonstrated strong associations with outcomes, with *R*^2^ values ranging from 10% to 15% for GFAP, NfL, Tau, UCH_L1, and S100B. For 6-month mortality, all the individual biomarkers, except for NSE, showed significant increases in *R*^2^ (ranging from 4–8% in the individual estimates). The composite biomarker also exhibited larger significant increases in *R*^2^ (17% for Core, 13% for Extended). For the 6-month poor recovery outcome, significant increases in *R*^2^ were found for GFAP, NfL, and Tau. The composite biomarker showed significantly larger increases in *R*^2^ (13% for Core, 9% for Extended), indicating a greater impact on the model fit. Although no individual biomarkers showed substantial differences in AUC when TRAJ clusters were added to the model, the composite biomarker did exhibit significant (*P* < 0.05) increases in AUC (0.07 for Core, 0.05 for Extended).

The enhanced performance for composite biomarkers is consistent with earlier findings (Tables 1 and 3, Fig. 3). It suggests that adding composite TRAJ clusters to a model with IMPACT does add value in improved fit (*R*^2^) for both outcomes and discriminative ability (AUC) for 6-month mortality. However, it is essential to note that the IMPACT scores are based solely on injury baseline information and do not include post-injury data collected over time. Comparing the TRAJ clusters based on the 5-day biomarker trajectories to the IMPACT collected at baseline is thus complicated by differences in information captured in the biomarkers as well as differences over time.

## Discussion

In this study, we examine the potential of biomarker trajectory patterns in predicting the outcomes of TBI patients at 3-, 6-, and 12-months post-injury. A previous study^8^ using the CENTER-TBI cohort evaluated the diagnostic and prognostic utility of GFAP, UCH-L1, NfL, Tau, S100B, and NSE measured within 24 h post-injury, examining their relationships with injury severity, care pathways, and the presence of CT abnormalities. A key finding was that all biomarkers were associated with TBI severity and outcomes, with GFAP showing the strongest ability to predict CT-positive findings across injury levels.^8^ To the best of our knowledge, the current study is the first to systematically examine six commonly used TBI biomarkers by trajectory clustering analysis among TBI patients in an ICU setting. Importantly, it is worth noting that the GCS scores at admission did not clearly distinguish the cluster groups (see Supplementary Fig. 2). GCS 3–8 (severe) accounts for 61–81% of the members of the high TRAJ class of the six biomarkers. Somewhat unexpectedly, GCS 3–8 still accounts for a sizable percentage (42–52% of members) in the low TRAJ class. This suggests that GCS scores are not the dominant factor driving the TRAJ classes. However, the clusters revealed significant differences in mortality rates, GOSE scores, and QoLIBRI-OS scores at follow-up intervals. Among the biomarkers, NSE showed the weakest performance, while the other five biomarkers consistently demonstrated significant associations between the trajectory class and the outcome measures.

An essential consideration in our modelling analysis is the number of trajectory (TRAJ) classes identified. As shown in Supplementary Table 3, our objectively derived model selection criteria consistently support a two-class solution as optimal for all six biomarkers, rather than three or more classes. However, in real-world scenarios, it is reasonable to expect that some patients may exhibit a delayed rise in biomarkers—potentially reflecting secondary injury processes—which could correspond to a yet undescribed trajectory class. Such a class may represent a small subgroup of patients. A key strength of the trajectory clustering model lies in its ability to balance the detection of latent subgroups based on temporal biomarker patterns with the model complexity. By identifying a manageable number of TRAJ classes with sufficient membership (e.g. >10–15%), the model enables meaningful subgroup analysis. This, in turn, allows further investigation into whether specific TRAJ classes are associated with good or poor clinical outcomes.

By focusing on the set of five biomarkers (GFAP, NfL, Tau, UCH-L1, and S100B), we observed substantial overlap between the low-trajectory and high-trajectory groups across these markers. We further identified 110 subjects who consistently belonged to the low-trajectory group for all five biomarkers (defined as the ‘Composite Biomarker [CB] Low TRAJ’ group), and 104 subjects who consistently belonged to the high-trajectory group across all five biomarkers (defined as the ‘CB High TRAJ’ group) (Supplementary Fig. 3). For simplicity, we refer to these groups as the ‘Low TRAJ’ and ‘High TRAJ’ classes, respectively. Notably, the strongest odds ratios for predicting poor and good outcomes were observed in the CB High TRAJ group and the CB Low TRAJ group, respectively, when compared to the corresponding odds ratios derived from individual biomarker trajectory groups. Adding the CB groups to a logistic regression model with IMPACT suggests that the 5-day trajectory clustering enhances the fit of a conventional model (based on baseline data) in predicting 6-month outcomes (Supplementary Table 5). Additionally, the CB groups based on 5 days of measurements consistently showed stronger, significantly different odds ratios compared to CB groups based on 1 day of measurements (Supplementary Table 5).

The incremental value was typically half or less as strong when adjusted for key baseline predictors as summarized in the IMPACT Core and Extended models. This finding highlights the importance of adjusting for baseline predictors when evaluating the value of biomarkers and their trajectories. NSE had less prognostic value. The composite biomarker performed best with unadjusted delta R^2^ values of 13% (mortality) and 17% (GOSE poor recovery), and with adjustment for the IMPACT Extended model (9%, GOSE poor recovery and 13%, mortality).

The incremental value analysis indicated that the TRAJ clusters provide added prognostic value, compared to the IMPACT prediction model, which serves as a baseline, incorporating only the limited information available at the initial time point. In contrast, the TRAJ clusters, derived from biomarker profiles, offer two primary advantages: they incorporate additional biological information and encompass a broader temporal window. This highlights the importance of clustering based on the trajectories of the 5-day repeated measurements of the serum biomarkers. The 5-day repeated measure model may account for post-day 1 adverse events such as secondary insults or brain injury, which would likely be a contributing factor to patient outcome.

The rationale for using biomarker trajectories across the acute and post-acute phases of TBI in ICU patients is that this approach is superior for tracking individuals who may exhibit persistently elevated biomarker levels over time, compared to relying on a single-point biomarker measurement (e.g. Day 1 sample). The findings inform that patients with high biomarker trajectories are more likely to experience a range of adverse outcomes, including increased mortality, poorer GOSE scores, and reduced quality of life. Thus, the novelty of this study lies in introducing a new paradigm—using repeated biomarker measurements over several days—to provide additional information and enhance our understanding of the patient’s disease course and trajectory.

To the best of our knowledge, this is only the second paper to discuss quality of life as a post-TBI metric in correlation with blood-based biomarker endpoints. Whitehouse *et al*.^67^ had examined initial blood-based biomarkers (≤ 24 h) and 6-month outcome among mild TBI subjects (GCS 13–15), taking into account QOLIBRI-OS. Since QoLIBRI-OS provides insight into the patient’s subjective experience of their well-being and daily functioning after TBI, it is encouraging to see that biomarker trajectory models can predict QoLIBRI-OS at 3 months post-injury (Table 4).

### Study limitations

This model is based solely on biomarker data without taking into account other patient information such as the intensity of the patients’ treatment or presence of intracranial surgery. The presence of missing biomarker data can result in imprecise model results; thus, our analysis is limited by the completeness of biomarker data collection and by relatively low numbers to define stable clusters. Moreover, the specific clustering approach impacts definition of clusters, such as the metric used to define the distance between data points. Another limitation is that the QoLIBRI-OS could not be completed by the most severely disabled patients, which is an inherent problem with collecting patient-reported outcomes in severely injured patients. Future studies involving larger cohorts will be needful to confirm our findings—particularly to determine whether the two-class TRAJ model remains optimal or if additional, smaller subclasses may emerge. In addition, this study did not include time points beyond day 5 post-injury or other TBI biomarkers such as phospho-Tau, puff-H, and inflammatory biomarkers such as IL-6.^13^

## Conclusion

This study demonstrates the clinical value of repeated measurement of serum biomarkers in gaining insight into various pathological processes within the post-traumatic brain injury brain and its recovery from injury. We showed that there is promise in constructing composite biomarkers to enhance the trajectory methods. For all biomarkers examined, as well as a composite biomarker model, the trajectory subclasses identified using 5-day repeated measures yield stronger odds ratios in outcome prediction when compared to their day 1-only counterparts. Thus, trajectory analysis may be a valuable way to integrate repeated measures of biomarker data and identify subgroups of patients who are at risk of poor outcomes versus those who can make a fuller recovery. Our findings suggest that the clinical utilization of TBI blood-based biomarkers in ICU settings should include repeat measures over time. The combination of biomarker data combined with trajectory profiling can help identify TBI patient subgroups characterized by significant differences in early and delayed mortality, global outcome, and quality of life endpoints.

## Supplementary Material

fcag055_Supplementary_Data

## Data Availability

The data used in this study were obtained from CENTER-TBI and stored on the Opal platform. (https://www.center-tbi.eu/data). Due to strict EU personal data protection regulations, individual-level data are not publicly available. Researchers interested in accessing the data may submit a detailed study proposal via this link: https://www.center-tbi.eu/data/study. Upon approval, data will made available under a data use agreement. All code used for the analyses is available on the Computational Learning Systems Lab GitHub repository: https://github.com/clslabMSU/Ensemble-Trajectory-Clustering-for-TBI-Biomarkers.
