# Supplementary material for: Serum biomarker trajectory clusters predict functional outcome and quality of life for traumatic brain injury
Source: Brain Commun. 2026 Feb 23;8(2):fcag055. doi: 10.1093/braincomms/fcag055 (PMC12970999; doi:10.1093/braincomms/fcag055)
Supplement: fcag055_Supplementary_Data [file fcag055_supplementary_data.pdf]

SUPPLEMENTARY MATERIALS

Serum biomarker trajectory clusters predict functional outcome and quality of life for traumatic brain injury

Thanh Son Do, Chantal Carnes, Zhihui Yang, Firas Kobeissy, Hamad Yadikar, Gayla Olbricht, Olli Tenovuo, Jussi P. Posti, Ewout W. Steyerberg, Lindsay Wilson, Nicole von Steinbüchel, Endre Czeiter, Andras Buki, David K. Menon, Andrew I.R. Maas, Kevin K. Wang*, Tayo Obafemi-Ajayi*, and the CENTER-TBI participants & Investigators^,

CENTER-TBI Investigators -

Cecilia Åkerlund[1], Krisztina Amrein[2], Nada Andelic[3], Lasse Andreassen[4], Audny Anke[5], Anna Antoni[6], Gérard Audibert[7], Philippe Azouvi[8], Maria Luisa Azzolini[9], Ronald Bartels[10], Pál Barzó[11], Romuald Beauvais[12], Ronny Beer[13], Bo-Michael Bellander[14], Antonio Belli[15], Habib Benali[16], Maurizio Berardino[17], Luigi Beretta[9], Morten Blaabjerg[18], Peter Bragge[19], Alexandra Brazinova[20], Vibeke Brinck[21], Joanne Brooker[22], Camilla Brorsson[23], Andras Buki[24], Monika Bullinger[25], Manuel Cabeleira[26], Alessio Caccioppola[27], Emiliana Calappi [27], Maria Rosa Calvi[9], Peter Cameron[28], Guillermo Carbayo Lozano[29], Marco Carbonara[27], Simona Cavallo[17], Giorgio Chevallard[30], Arturo Chieregato[30], Giuseppe Citerio[31, 32], Hans Clusmann[33], Mark Coburn[34], Jonathan Coles[35], Jamie D. Cooper[36], Marta Correia[37], Amra Čović [38], Nicola Curry[39], Endre Czeiter[24], Marek Czosnyka[26], Claire Dahyot-Fizelier[40], Paul Dark[41], Helen Dawes[42], Véronique De Keyser[43], Vincent Degos[16], Francesco Della Corte[44], Hugo den Boogert[10], Bart Depreitere[45], Đula Đilvesi [46], Abhishek Dixit[47], Emma Donoghue[22], Jens Dreier[48], Guy-Loup Dulière[49], Ari Ercole[47], Patrick Esser[42], Erzsébet Ezer[50], Martin Fabricius[51], Valery L. Feigin[52], Kelly Foks[53], Shirin Frisvold[54], Alex Furmanov[55], Pablo Gagliardo[56], Damien Galanaud[16], Dashiell Gantner[28], Guoyi Gao[57], Pradeep George[58], Alexandre Ghuysen[59], Lelde Giga[60], Ben Glocker[61], Jagoš Golubovic[46], Pedro A. Gomez [62], Johannes Gratz[63], Benjamin Gravesteijn[64], Francesca Grossi[44], Russell L. Gruen[65], Deepak Gupta[66], Juanita A. Haagsma[64], Iain Haitsma[67], Raimund Helbok[68,69], Eirik Helseth[70], Lindsay Horton [71], Jilske Huijben[64], Peter J. Hutchinson[72], Bram Jacobs[73], Stefan Jankowski[74], Mike Jarrett[21], Ji-yao Jiang[58], Faye Johnson[75], Kelly Jones[52], Mladen Karan[46], Angelos G. Kolias[72], Erwin Kompanje[76], Daniel Kondziella[51], Evgenios Kornaropoulos[47], Lars-Owe Koskinen[77], Noémi Kovács[78], Ana Kowark[79], Alfonso Lagares[62], Linda Lanyon[58], Steven Laureys[80], Fiona Lecky[81, 82], Didier Ledoux[80], Rolf Lefering[83], Valerie Legrand[84], Aurelie Lejeune[85], Leon Levi[86], Roger Lightfoot[87], Hester Lingsma[64],

Andrew I.R. Maas[43,88], Ana M. Castaño-León[62], Marc Maegele[89], Marek Majdan[20], Alex Manara[90], Geoffrey Manley[91], Costanza Martino[92], Hugues Maréchal[49], Julia Mattern[93], Catherine McMahon[94], Béla Melegh[95], David Menon[47], Tomas Menovsky[43,88], Ana Mikolic[64], Benoit Misset[80], Visakh Muraleedharan[58], Lynnette Murray[28], Ancuta Negru[96], David Nelson[1], Virginia Newcombe[47], Daan Nieboer[64], József Nyirádi[2], Otesile Olubukola[81], Matej Oresic[97], Fabrizio Ortolano[27], Aarno Palotie[98, 99, 100], Paul M. Parizel[101], Jean-François Payen[102], Natascha Perera[12], Vincent Perlbarg[16], Paolo Persona[103], Wilco Peul[104], Anna Piippo-Karjalainen[105], Matti Pirinen[98], Dana Pisica[64], Horia Ples[96], Suzanne Polinder[64], Inigo Pomposo[29], Jussi P. Posti [106], Louis Puybasset[107], Andreea Radoi [108], Arminas Ragauskas[109], Rahul Raj[105], Malinka Rambadagalla[110], Isabel Retel Helmrich[64], Jonathan Rhodes[111], Sylvia Richardson[112], Sophie Richter[47], Samuli Ripatti[98], Saulius Rocka[109], Cecilie Roe[113], Olav Roise[114,115], Jonathan Rosand[116], Jeffrey V. Rosenfeld[117], Christina Rosenlund[118], Guy Rosenthal[55], Rolf Rossaint[79], Sandra Rossi[103], Daniel Rueckert[61] Martin Rusnák[119], Juan Sahuquillo[108], Oliver Sakowitz[93, 120], Renan Sanchez-Porras[120], Janos Sandor[121], Nadine Schäfer[83], Silke Schmidt[122], Herbert Schoechl[123], Guus Schoonman[124], Rico Frederik Schou[125], Elisabeth Schwendenwein[6], Charlie Sewalt[64], Ranjit D. Singh[104], Toril Skandsen[126, 127] , Peter Smielewski[26], Abayomi Sorinola[128], Emmanuel Stamatakis[47], Simon Stanworth[39], Robert Stevens[129], William Stewart[130], Ewout W. Steyerberg[64, 131], Nino Stocchetti[132], Nina Sundström[133], Riikka Takala[134], Viktória Tamás[128], Tomas Tamosuitis[135], Mark Steven Taylor[20], Aurore Thibaut[80], Braden Te Ao[52], Olli Tenovuo[106], Alice Theadom[52], Matt Thomas[90], Dick Tibboel[136], Marjolein Timmers[76], Christos Tolias[137], Tony Trapani[28], Cristina Maria Tudora[96], Andreas Unterberg[93], Peter Vajkoczy [138], Shirley Vallance[28], Egils Valeinis[60], Zoltán Vámos[50], Mathieu van der Jagt[139], Gregory Van der Steen[43], Joukje van der Naalt[73], Jeroen T.J.M. van Dijck [104], Inge A. M. van Erp[104], Thomas A. van Essen[104], Wim Van Hecke[140], Caroline van Heugten[141], Ernest van Veen[64], Thijs Vande Vyvere[142], Roel P. J. van Wijk[104], Alessia Vargiolu[32], Emmanuel Vega[85], Kimberley Velt[64], Jan Verheyden[140], Paul M. Vespa[143], Anne Vik[126, 144], Rimantas Vilcinis[135], Victor Volovici[67], Nicole von Steinbüchel[38], Daphne Voormolen[64], Petar Vulekovic[46], Kevin K.W. Wang[145], Daniel Whitehouse[47], Eveline Wiegers[64], Guy Williams[47], Lindsay Wilson[71], Stefan Winzeck[47], Stefan Wolf[146], Zhihui Yang[116], Peter Ylén[147], Alexander Younsi[93], Frederick A. Zeiler[47,148], Veronika Zelinkova[20], Agate Ziverte[60] , Tommaso Zoerle[27]

[1] Department of Physiology and Pharmacology, Section of Perioperative Medicine and Intensive Care, Karolinska Institutet, Stockholm, Sweden

[2] János Szentágothai Research Centre, University of Pécs, Pécs, Hungary

[3] Division of Clinical Neuroscience, Department of Physical Medicine and Rehabilitation, Oslo University Hospital and University of Oslo, Oslo, Norway

[4] Department of Neurosurgery, University Hospital Northern Norway, Tromso, Norway

[5] Department of Physical Medicine and Rehabilitation, University Hospital Northern Norway, Tromso, Norway

[6] Trauma Surgery, Medical University Vienna, Vienna, Austria

[7] Department of Anesthesiology & Intensive Care, University Hospital Nancy, Nancy, France

[8] Raymond Poincare hospital, Assistance Publique – Hopitaux de Paris, Paris, France

[9] Department of Anesthesiology & Intensive Care, S Raffaele University Hospital, Milan, Italy

[10] Department of Neurosurgery, Radboud University Medical Center, Nijmegen, The Netherlands

[11] Department of Neurosurgery, University of Szeged, Szeged, Hungary

[12] International Projects Management, ARTTIC, Munchen, Germany

[13] Department of Neurology, Neurological Intensive Care Unit, Medical University of Innsbruck, Innsbruck, Austria

[14] Department of Neurosurgery & Anesthesia & intensive care medicine, Karolinska University Hospital, Stockholm, Sweden

[15] NIHR Surgical Reconstruction and Microbiology Research Centre, Birmingham, UK

[16] Anesthesie-Réanimation, Assistance Publique – Hopitaux de Paris, Paris, France

[17] Department of Anesthesia & ICU, AOU Città della Salute e della Scienza di Torino - Orthopedic and Trauma Center, Torino, Italy

[18] Department of Neurology, Odense University Hospital, Odense, Denmark

[19] BehaviourWorks Australia, Monash Sustainability Institute, Monash University, Victoria, Australia

[20] Department of Public Health, Faculty of Health Sciences and Social Work, Trnava University, Trnava, Slovakia

[21] Quesgen Systems Inc., Burlingame, California, USA

[22] Australian & New Zealand Intensive Care Research Centre, Department of Epidemiology and Preventive Medicine, School of Public Health and Preventive Medicine, Monash University, Melbourne, Australia

[23] Department of Surgery and Perioperative Science, Umeå University, Umeå, Sweden

[24] Department of Neurosurgery, Medical School, University of Pécs, Hungary and Neurotrauma Research Group, János Szentágothai Research Centre, University of Pécs, Hungary

[25] Department of Medical Psychology, Universitätsklinikum Hamburg-Eppendorf, Hamburg, Germany

[26] Brain Physics Lab, Division of Neurosurgery, Dept of Clinical Neurosciences, University of Cambridge, Addenbrooke's Hospital, Cambridge, UK

[27] Neuro ICU, Fondazione IRCCS Cà Granda Ospedale Maggiore Policlinico, Milan, Italy

[28] ANZIC Research Centre, Monash University, Department of Epidemiology and Preventive Medicine, Melbourne, Victoria, Australia

[29] Department of Neurosurgery, Hospital of Cruces, Bilbao, Spain

[30] NeuroIntensive Care, Niguarda Hospital, Milan, Italy

[31] School of Medicine and Surgery, Università Milano Bicocca, Milano, Italy

[32] NeuroIntensive Care Unit, Department Neuroscience, IRCCS Fondazione San Gerardo dei Tintori, Monza, Italy

[33]Department of Neurosurgery, Medical Faculty RWTH Aachen University, Aachen, Germany

[34] Department of Anesthesiology and Intensive Care Medicine, University Hospital Bonn, Bonn, Germany

[35] Department of Anesthesia & Neurointensive Care, Cambridge University Hospital NHS Foundation Trust, Cambridge, UK

[36] School of Public Health & PM, Monash University and The Alfred Hospital, Melbourne, Victoria, Australia

[37] Radiology/MRI department, MRC Cognition and Brain Sciences Unit, Cambridge, UK

[38] Institute of Medical Psychology and Medical Sociology, Universitätsmedizin Göttingen, Göttingen, Germany

[39] Oxford University Hospitals NHS Trust, Oxford, UK

[40] Intensive Care Unit, CHU Poitiers, Potiers, France

[41] University of Manchester NIHR Biomedical Research Centre, Critical Care Directorate, Salford Royal Hospital NHS Foundation Trust, Salford, UK

[42] Movement Science Group, Faculty of Health and Life Sciences, Oxford Brookes University, Oxford, UK

[43] Department of Neurosurgery, Antwerp University Hospital, Edegem, Belgium

[44] Department of Anesthesia & Intensive Care, Maggiore Della Carità Hospital, Novara, Italy

[45] Department of Neurosurgery, University Hospitals Leuven, Leuven, Belgium

[46] Department of Neurosurgery, Clinical centre of Vojvodina, Faculty of Medicine, University of Novi Sad, Novi Sad, Serbia

[47] Division of Anaesthesia, University of Cambridge, Addenbrooke's Hospital, Cambridge, UK

[48] Center for Stroke Research Berlin, Charité – Universitätsmedizin Berlin, corporate member of Freie Universität Berlin, Humboldt-Universität zu Berlin, and Berlin Institute of Health, Berlin, Germany

[49] Intensive Care Unit, CHR Citadelle, Liège, Belgium

[50] Department of Anaesthesiology and Intensive Therapy, University of Pécs, Pécs, Hungary

[51] Departments of Neurology, Clinical Neurophysiology and Neuroanesthesiology, Region Hovedstaden Rigshospitalet, Copenhagen, Denmark

[52] National Institute for Stroke and Applied Neurosciences, Faculty of Health and Environmental Studies, Auckland University of Technology, Auckland, New Zealand

[53] Department of Neurology, Erasmus MC, Rotterdam, the Netherlands

[54] Department of Anesthesiology and Intensive care, University Hospital Northern Norway, Tromso, Norway

[55] Department of Neurosurgery, Hadassah-hebrew University Medical center, Jerusalem, Israel

[56] Fundación Instituto Valenciano de Neurorrehabilitación (FIVAN), Valencia, Spain

[57] Department of Neurosurgery, Shanghai Renji hospital, Shanghai Jiaotong University/school of medicine, Shanghai, China

[58] Karolinska Institutet, INCF International Neuroinformatics Coordinating Facility, Stockholm, Sweden

[59] Emergency Department, CHU, Liège, Belgium

[60] Neurosurgery clinic, Pauls Stradins Clinical University Hospital, Riga, Latvia

[61] Department of Computing, Imperial College London, London, UK

[62] Department of Neurosurgery, Hospital Universitario 12 de Octubre, Madrid, Spain

[63] Department of Anesthesia, Critical Care and Pain Medicine, Medical University of Vienna, Austria

[64] Department of Public Health, Erasmus Medical Center-University Medical Center, Rotterdam, The Netherlands

[65] College of Health and Medicine, Australian National University, Canberra, Australia

[66] Department of Neurosurgery, Neurosciences Centre & JPN Apex trauma centre, All India Institute of Medical Sciences, New Delhi-110029, India

[67] Department of Neurosurgery, Erasmus MC, Rotterdam, the Netherlands

[68] Department of Neurology, Kepler University Hospital, Johannes Kepler University Linz, Linz, Austria.

[69] Clinical Research Institute for Neuroscience, Johannes Kepler University Linz, Linz, Austria

[70] Department of Neurosurgery, Oslo University Hospital, Oslo, Norway

[71] Division of Psychology, University of Stirling, Stirling, UK

[72] Division of Neurosurgery, Department of Clinical Neurosciences, Addenbrooke's Hospital & University of Cambridge, Cambridge, UK

[73] Department of Neurology, University of Groningen, University Medical Center Groningen, Groningen, Netherlands

[74] Neurointensive Care , Sheffield Teaching Hospitals NHS Foundation Trust, Sheffield, UK

[75] Salford Royal Hospital NHS Foundation Trust Acute Research Delivery Team, Salford, UK

[76] Department of Intensive Care and Department of Ethics and Philosophy of Medicine, Erasmus Medical Center, Rotterdam, The Netherlands

[77] Department of Clinical Neuroscience, Neurosurgery, Umeå University, Umeå, Sweden

[78] Hungarian Brain Research Program - Grant No. KTIA_13_NAP-A-II/8, University of Pécs, Pécs, Hungary

[79] Department of Anaesthesiology, University Hospital of Aachen, Aachen, Germany

[80] Cyclotron Research Center , University of Liège, Liège, Belgium

[81] Centre for Urgent and Emergency Care Research (CURE), Health Services Research Section, School of Health and Related Research (ScHARR), University of Sheffield, Sheffield, UK

[82] Emergency Department, Salford Royal Hospital, Salford UK

[83] Institute of Research in Operative Medicine (IFOM), Witten/Herdecke University, Cologne, Germany

[84] VP Global Project Management CNS, ICON, Paris, France

[85] Department of Anesthesiology-Intensive Care, Lille University Hospital, Lille, France

[86] Department of Neurosurgery, Rambam Medical Center, Haifa, Israel

[87] Department of Anesthesiology & Intensive Care, University Hospitals Southhampton NHS Trust, Southhampton, UK

[88] Department of Translational Neuroscience, Faculty of Medicine and Health Science, University of Antwerp, Antwerp, Belgium

[89] Cologne-Merheim Medical Center (CMMC), Department of Traumatology, Orthopedic Surgery and Sportmedicine, Witten/Herdecke University, Cologne, Germany

[90] Intensive Care Unit, Southmead Hospital, Bristol, Bristol, UK

[91] Department of Neurological Surgery, University of California, San Francisco, California, USA

[92] Department of Anesthesia & Intensive Care,M. Bufalini Hospital, Cesena, Italy

[93] Department of Neurosurgery, University Hospital Heidelberg, Heidelberg, Germany

[94] Department of Neurosurgery, The Walton centre NHS Foundation Trust, Liverpool, UK

[95] Department of Medical Genetics, University of Pécs, Pécs, Hungary

[96] Department of Neurosurgery, Emergency County Hospital Timisoara , Timisoara, Romania

[97] School of Medical Sciences, Örebro University, Örebro, Sweden

[98] Institute for Molecular Medicine Finland, University of Helsinki, Helsinki, Finland

[99] Analytic and Translational Genetics Unit, Department of Medicine; Psychiatric & Neurodevelopmental Genetics Unit, Department of Psychiatry; Department of Neurology, Massachusetts General Hospital, Boston, MA, USA

[100] Program in Medical and Population Genetics; The Stanley Center for Psychiatric Research, The Broad Institute of MIT and Harvard, Cambridge, MA, USA

[101] Department of Radiology, University of Antwerp, Edegem, Belgium

[102] Department of Anesthesiology & Intensive Care, University Hospital of Grenoble, Grenoble, France

[103] Department of Anesthesia & Intensive Care, Azienda Ospedaliera Università di Padova, Padova, Italy

[104] Dept. of Neurosurgery, Leiden University Medical Center, Leiden, The Netherlands and Dept. of Neurosurgery, Medical Center Haaglanden, The Hague, The Netherlands

[105] Department of Neurosurgery, Helsinki University Central Hospital

[106] Division of Clinical Neurosciences, Department of Neurosurgery and Turku Brain Injury Centre, Turku University Hospital and University of Turku, Turku, Finland

[107] Department of Anesthesiology and Critical Care, Pitié -Salpêtrière Teaching Hospital, Assistance Publique, Hôpitaux de Paris and University Pierre et Marie Curie, Paris, France

[108] Neurotraumatology and Neurosurgery Research Unit (UNINN), Vall d'Hebron Research Institute, Barcelona, Spain

[109] Department of Neurosurgery, Kaunas University of technology and Vilnius University, Vilnius, Lithuania

[110] Department of Neurosurgery, Rezekne Hospital, Latvia

[111] Department of Anaesthesia, Critical Care & Pain Medicine NHS Lothian & University of Edinburg, Edinburgh, UK

[112] Director, MRC Biostatistics Unit, Cambridge Institute of Public Health, Cambridge, UK

[113] Department of Physical Medicine and Rehabilitation, Oslo University Hospital/University of Oslo, Oslo, Norway

[114] Division of Orthopedics, Oslo University Hospital, Oslo, Norway

[115] Institue of Clinical Medicine, Faculty of Medicine, University of Oslo, Oslo, Norway

[116] Broad Institute, Cambridge MA Harvard Medical School, Boston MA, Massachusetts General Hospital, Boston MA, USA

[117] National Trauma Research Institute, The Alfred Hospital, Monash University, Melbourne, Victoria, Australia

[118] Department of Neurosurgery, Odense University Hospital, Odense, Denmark

[119] International Neurotrauma Research Organisation, Vienna, Austria

[120] Klinik für Neurochirurgie, Klinikum Ludwigsburg, Ludwigsburg, Germany

[121] Division of Biostatistics and Epidemiology, Department of Preventive Medicine, University of Debrecen, Debrecen, Hungary

[122] Department Health and Prevention, University Greifswald, Greifswald, Germany

[123] Department of Anaesthesiology and Intensive Care, AUVA Trauma Hospital, Salzburg, Austria

[124] Department of Neurology, Elisabeth-TweeSteden Ziekenhuis, Tilburg, the Netherlands

[125] Department of Neuroanesthesia and Neurointensive Care, Odense University Hospital, Odense, Denmark

[126] Department of Neuromedicine and Movement Science, Norwegian University of Science and Technology, NTNU, Trondheim, Norway

[127] Department of Physical Medicine and Rehabilitation, St.Olavs Hospital, Trondheim University Hospital, Trondheim, Norway

[128] Department of Neurosurgery, University of Pécs, Pécs, Hungary

[129] Division of Neuroscience Critical Care, John Hopkins University School of Medicine, Baltimore, USA

[130] Department of Neuropathology, Queen Elizabeth University Hospital and University of Glasgow, Glasgow, UK

[131] Dept. of Department of Biomedical Data Sciences, Leiden University Medical Center, Leiden, The Netherlands

[132] Department of Pathophysiology and Transplantation, Milan University, and Neuroscience ICU, Fondazione IRCCS Cà Granda Ospedale Maggiore Policlinico, Milano, Italy

[133] Department of Radiation Sciences, Biomedical Engineering, Umeå University, Umeå, Sweden

[134] Perioperative Services, Intensive Care Medicine and Pain Management, Turku University Hospital and University of Turku, Turku, Finland

[135] Department of Neurosurgery, Kaunas University of Health Sciences, Kaunas, Lithuania

[136] Intensive Care and Department of Pediatric Surgery, Erasmus Medical Center, Sophia Children's Hospital, Rotterdam, The Netherlands

[137] Department of Neurosurgery, Kings college London, London, UK

[138] Neurologie, Neurochirurgie und Psychiatrie, Charité – Universitätsmedizin Berlin, Berlin, Germany

[139] Department of Intensive Care Adults, Erasmus MC– University Medical Center Rotterdam, Rotterdam, the Netherlands

[140] icoMetrix NV, Leuven, Belgium

[141] Movement Science Group, Faculty of Health and Life Sciences, Oxford Brookes University, Oxford, UK

[142] Radiology Department, Antwerp University Hospital and University of Antwerp, Edegem, Belgium

[143] Director of Neurocritical Care, University of California, Los Angeles, USA

[144] Department of Neurosurgery, St.Olavs Hospital, Trondheim University Hospital, Trondheim, Norway

[145] Department of Emergency Medicine, University of Florida, Gainesville, Florida, USA

[146] Department of Neurosurgery, Charité – Universitätsmedizin Berlin, corporate member of Freie Universität Berlin, Humboldt-Universität zu Berlin, and Berlin Institute of Health, Berlin, Germany

[147] VTT Technical Research Centre, Tampere, Finland

[148] Section of Neurosurgery, Department of Surgery, Rady Faculty of Health Sciences, University of Manitoba, Winnipeg, MB, Canada

Clustering Algorithms

The k-means algorithm is one of the most popular partitional clustering algorithms; it is regarded as a staple of clustering methods because of its merits and influence on other clustering approaches. It is based on an iterative optimization procedure to seek an optimal K-partition of data by minimizing the sum-of-squared-error criterion. Spectral clustering constructs a similarity graph on the data and derives the clusters by partitioning the embedding of the graph Laplacian. Agglomerative clustering is a type of hierarchical clustering based on a bottom-up approach. It initially assigns each data point to a cluster and then iteratively merges the clusters based on proximity (or linkage) measures, until a stopping criterion is attained. Gaussian mixture clustering determines the number of components in a mixture and estimates the parameters of each component based on the observations. Sequential k-means is a modification of the k-means algorithm to cluster sequential data by using the Kullback-Leibler distance rather than the Euclidean distance. For each of the algorithms, the number of clusters (k) is set a priori. In this work, we varied k from 2 to 5, given the relatively small number of samples.

Supplementry Table 1 Missing values distribution per biomarker across the first 5 days post injury.

Biomarker	Day 1	Day 2	Day 3	Day 4	Day 5
GFAP (373)	1 (0.27%)	27 (7.24%)	69 (18.50%)	89 (23.86%)	122 (32.71%)
NfL (373)	1 (0.27%)	28 (7.51%)	73 (19.57%)	91 (24.40%)	126 (33.78%)
Tau (373)	0 (0.00%)	27 (7.24%)	73 (19.57%)	93 (24.93%)	126 (33.78%)
UCH-L1(373)	1 (0.27%)	28 (7.51%)	77 (20.64%)	95 (25.47%)	128 (34.32%)
S100B (373)	1 (0.27%)	24 (6.43%)	65 (17.43%)	91 (24.40%)	121 (32.44%)
NSE (373)	2 (0.54%)	24 (6.43%)	65 (17.43%)	91 (24.40%)	121 (32.44%)

Supplementary Table 2 Internal validation metrics for ensemble cluster validation model

Silhouette index (Sil)	$\frac{1}{k}\sum_{i=1}^{k}\left[\frac{1}{n_i}\sum_{x\in C_i}\frac{b(x)-a(x)}{max_x\big(b(x),a(x)\big)}\right]$ Where: $a(x)=\frac{1}{n_i-1}\sum_{y\in C_i,y\neq x}d(x,y)$ $b(x)=min_{j,j\neq i}\left[\frac{1}{n_j}\sum_{y\in C_j}d(x,y)\right]$
Mean Index Accuracy (MIA)	$\sqrt{\frac{1}{k}\sum_{i=1}^{\mathrm{k}}\sum_{x\in C_i}d^2(x,c_i)}$
Similarity Matrix Index (SMI)	$\mathrm{max}_{i>j}\left[\left(1-\frac{1}{ln\left(d(c_i,c_j)\right)}\right)^{-1}\right]$
Davies-Bouldin index (DB)	$\frac{1}{k}\sum_{i=1}^{k}max_{j,j\neq i}\left[\frac{\frac{1}{n_i}sum_{x\in C_i}d(x,c_i)\ +\ \frac{1}{n_j}sum_{x\in C_j}d(x,c_j)}{d(c_i,c_j)}\right]$
Xie-Beni index (XB)	$\frac{\sum_{i=1}^{k}\sum_{x\in C_i}d^2(x,c_i)}{N\ min_{i,i\neq j}d^2(c_i,c_j)}$
Calinski-Harabasz index (CH)	$\frac{\sum_i n_i\ d^2(c_i,c)/(k-1)}{\sum_i\sum_{x\in C_i}d^2(x,c_i)/(N-k)}$
CVNN validation index based on nearest neighbors	$Sep_{norm}(k,knn)+Com_{norm}(k)$ Where Sep is an inter-cluster Separation function: $\mathrm{Sep}_{norm}(k,knn)=\frac{Sep(k,knn)}{max_{C\subset D}Sep(k,knn)}$ $Sep(k,knn)=max_{i=1,2,\ldots k}\left[\frac{1}{n_i}\sum_{j=1..n_i}\left(\frac{q_j}{k}\right)\right]$ And Com is an intra-cluster compactness function: $Com_{norm}(k)=\frac{Com(k)}{max_{C\subset D}Com(k)}$ $Com(k)=\sum_{\mathrm{i}}\left[\frac{2}{\mathrm{n_i(n_i-1)}}\sum_{\mathrm{x,y}\in C_{\mathrm{i}}}\mathrm{d(x,y)}\right]$

D: data set; n: number of objects in D; cluster C: center of D; k: number of clusters; C_i: the i–th cluster; n_i: number of objects in cluster C_i; c_i: center of cluster C_i; $\mathrm{d(x,y)}$: distance between x and y; knn: number of nearest neighbors; q_j: is the number of nearest neighbors of C_j not in cluster C_i

Supplementary Table 3 Top five optimal results of ensemble cluster validation model by biomarker.

		Sil	Db	XB	CH	CVNN	MIA	SMI	Total
**GFAP**									
	ag_k2	5	1	5	4	0	0	5	20
	sp_k2	4	2	4	1	0	0	4	15
	km_k2	3	3	3	0	0	0	3	12
	sp_k3	0	5	1	5	0	0	0	11
	gm_k3	0	0	2	0	2	0	2	6
**NFL**									
	ag_k2	4	1	5	5	0	0	5	20
	gm_k2	5	0	4	0	1	0	4	14
	km_k2	3	2	3	0	0	0	3	11
	sp_k2	0	5	2	0	0	0	2	9
	sp_k3	0	4	0	4	0	0	0	8
**Tau**									
	sp_k2	4	4	5	5	0	0	4	22
	km_k2	3	5	4	4	0	0	3	19
	ag_k2	2	2	3	2	0	0	2	11
	gm_k5	0	0	0	0	4	2	0	6
	km_k3	1	0	2	3	0	0	0	6
**UCH-L1**									
	km_k2	3	3	5	4	0	0	5	20
	sp_k2	1	4	4	2	0	0	4	15
	ag_k2	0	5	3	0	0	0	3	11
	gm_k4	2	0	2	0	5	0	1	10
	gm_k2	5	0	1	0	0	0	2	8
**S100B**									
	km_k2	4	5	5	4	0	0	5	23
	sp_k2	3	4	4	3	0	0	4	18
	km_k3	2	2	2	5	0	0	1	12
	ag_k2	0	3	3	0	0	0	3	9
	gm_k2	5	0	0	0	0	0	2	7
**NSE**									
	ag_k2	5	5	5	5	0	0	4	24
	km_k2	3	1	4	3	0	0	3	14
	gm_k2	4	0	3	0	0	0	2	9
	km_k3	1	3	1	4	0	0	0	9
	gm_k5	0	0	0	0	5	2	0	7

Algorithms: k-means- *km*, spectral- *sp*, agglomerate- *ag*, gaussian mixture *gm*, and sequential kmeans- *sk*. Each clustering outcome is varied from 2 to 5 clusters (*k2* to *k5*) and evaluated using internal cluster validation metrics.

Supplementary Table 4 Statistical comparisons of clusters based on day1-5 vs. day 1 biomarker data using the Breslow-Day test

	Low TRAJ/ good GOSE (7-8 - True)			High TRAJ/ poor GOSE (1-4 - True)		
**Timepoint** (month)	**5 Days OR (95%CI)**	**Day 1 OR (95%CI)**	**Breslow-Day p-value**	**5 Days OR (95%CI)**	**Day 1 OR (95%CI)**	**Breslow-Day p-value**
**GFAP clusters**						
3	5.92 (2.67 to 13.1) †††	3.41 (1.67, 6.96)†††	0.31	3.75 (2.3 to 6.12) †††	2.87 (1.77, 4.64)†††	0.44
6	3.75 (2.08 to 6.74) †††	2.3 (1.31, 4.01)††	0.24	3.58 (2.29 to 5.6) †††	2.55 (1.65, 3.96)†††	0.29
12	3.11 (1.88 to 5.15) †††	1.69 (1.04, 2.75)†	0.09	4.58 (2.9 to 7.24) †††	2.54 (1.64, 3.94)†††	0.07
**NFL clusters**						
3	5.87 (2.42 to 14.25) †††	2.81 (1.38, 5.74)††	0.20	5.69 (3.26 to 9.96) †††	2.37 (1.46, 3.85)†††	**0.02**
6	4.52 (2.32 to 8.8) †††	2.17 (1.23, 3.84)††	0.10	4.63 (2.89 to 7.41) †††	2.46 (1.58, 3.83)†††	0.05
12	4.82 (2.69 to 8.61) †††	2.36 (1.42, 3.93)†††	0.07	5.01 (3.15 to 7.98) †††	2.45 (1.58, 3.8)†††	**0.03**
**Tau clusters**						
3	4.61 (2.08 to 10.21) †††	4.28 (2.16, 8.46)†††	0.89	3.49 (2.11 to 5.76) †††	3.03 (1.9, 4.84)†††	0.69
6	3.14 (1.73 to 5.7) †††	2.6 (1.51, 4.46)†††	0.64	3.75 (2.38 to 5.9) †††	2.03 (1.31, 3.14)††	0.06
12	3.26 (1.93 to 5.52) †††	2.49 (1.53, 4.04)†††	0.46	4.46 (2.83 to 7.05) †††	2.26 (1.46, 3.52)†††	**0.04**
**UCH-L1 clusters**						
3	5.45 (2.75 to 10.82) †††	3.65 (1.79, 7.46)†††	0.43	3.56 (2.21 to 5.72) †††	2.79 (1.73, 4.49)†††	0.48
6	2.99 (1.74 to 5.13) †††	2.51 (1.44, 4.39)††	0.66	3.13 (1.98 to 4.94) †††	2.79 (1.8, 4.33)†††	0.73
12	3.06 (1.88 to 4.99) †††	2.55 (1.55, 4.2)†††	0.61	4.92 (3.01 to 8.05) †††	2.57 (1.66, 3.98)†††	**0.05**
**S100B clusters**						
3	4.82 (2.31 to 10.05) †††	1.55 (0.79, 3.02)	**0.02**	4.28 (2.62 to 6.99) †††	2.29 (1.39, 3.77)††	0.08
6	2.96 (1.69 to 5.18) †††	1.37 (0.79, 2.39)	0.06	3.84 (2.45 to 6.02) †††	1.8 (1.15, 2.8)†	**0.02**
12	2.87 (1.75 to 4.72) †††	1.44 (0.87, 2.37)	**0.05**	4.32 (2.73 to 6.82) †††	2.1 (1.35, 3.27)††	0.03
**NSE clusters**						
3	2.69 (0.62 to 11.65)	2.65 (1.27, 5.53)††	0.98	3.99 (1.37 to 11.66) †	2.23 (1.36, 3.65)††	0.33
6	4.39 (1.02 to 18.82)	1.76 (0.99, 3.12)	0.24	3.12 (1.37 to 7.14) ††	1.33 (0.86, 2.06)	0.07
12	14.06 (1.89 to 104.39) ††	**1.27 (0.77, 2.07)**	**0.01**	3.46 (1.56 to 7.69) ††	1.55 (1.0, 2.41)	0.08
**Composite Biomarker (CB) clusters**						
3	10.83 (3.65 to 32.14) †††	4.13 (2.06 to 8.29) †††	0.14	10.49 (4.96 to 22.18) †††	3.08 (1.92 to 4.94) †††	**0.006**
6	7.42 (3.10 to 17.76) †††	2.4 (1.4 to 4.13) ††	**0.03**	8.79 (4.56 to 16.97) †††	2.48 (1.6 to 3.84) †††	**0.001**
12	7.99 (3.71 to 17.20) †††	2.1 (1.3 to 3.39) ††	**0.003**	12.29 (6.19 to 24.40) †††	2.68 (1.72 to 4.18) †††	**0.0002**

Testing the differences in the odds ratios between optimal clustering model configuration outcomes based on day1- 5 vs. day 1 biomarker data using the Breslow-Day test
with p-values reported. Those that are statistically significant at $\alpha=0.05$ are in bold. † $P < 0.05$, †† $p < 0.01$, ††† $p<0.001$

Supplementary Table 5 Incremental value analysis of 5 day biomarker TRAJ cluster over IMPACT model

Biomarker	Univariate R^2	IMPACT R^2	Delta R^2 (95% CI)	Univariate AUC	IMPACT AUC	Delta AUC (95%CI)
Core model, 6-month poor recovery GOSE outcome (1-4)						
Composite (CB)	0.32	0.34	0.13/ (0.04- 0.23)†	0.75	0.79	0.07/ (0.01- 0.1)†
GFAP	0.14	0.36	0.05/ (0.01- 0.1)†	0.66	0.80	0.02/ (0- 0.05)†
NfL	0.18	0.36	0.08/ (0.03- 0.14)†	0.69	0.80	0.04/ (0.01- 0.06)†
Tau	0.14	0.36	0.06/ (0.02- 0.12)†	0.67	0.80	0.03/ (0.01- 0.05)†
UCH-L1	0.12	0.36	0.04/ (0.01- 0.09)†	0.65	0.80	0.02/ (0- 0.04)†
S100B	0.15	0.36	0.04/ (0.01- 0.09)†	0.67	0.80	0.02/ (0- 0.04)†
NSE	0.04	0.36	0.01/ (0- 0.05)†	0.55	0.80	0.01/ (0- 0.02)†
Core model, 6-month mortality						
Composite (CB)	0.28	0.36	0.17/ (0.06- 0.27)†	0.76	0.71	0.09/ (0.02- 0.13)†
GFAP	0.11	0.32	0.04/ (0.01- 0.1)†	0.67	0.73	0.03/ (0- 0.05)†
NfL	0.14	0.32	0.1/ (0.04- 0.18)†	0.69	0.73	0.06/ (0.02- 0.09)†
Tau	0.13	0.32	0.08/ (0.03- 0.15)†	0.68	0.73	0.04/ (0.01- 0.07)†
UCH-L1	0.11	0.32	0.06/ (0.02- 0.12)†	0.66	0.73	0.04/ (0- 0.06)†
S100B	0.17	0.32	0.08/ (0.03- 0.14)†	0.70	0.73	0.03/ (0- 0.06)†
NSE	0.07	0.32	0.05/ (0.01- 0.13)†	0.58	0.73	0.01/ (-0.01- 0.02)
Extended model, 6-month poor recovery GOSE outcome (1-4)						
Composite (CB)	0.32	0.44	0.09/ (0.02- 0.17)†	0.75	0.83	0.05/ (0.01- 0.07)†
GFAP	0.14	0.42	0.03/ (0.01- 0.08)†	0.66	0.83	0.01/ (0- 0.03)†
NfL	0.18	0.42	0.06/ (0.02- 0.12)†	0.69	0.83	0.03/ (0.01- 0.05)†
Tau	0.14	0.42	0.05/ (0.01- 0.1)†	0.67	0.83	0.02/ (0- 0.04)†
UCH-L1	0.12	0.42	0.02/ (0- 0.07)†	0.65	0.83	0.01/ (0- 0.03)†
S100B	0.15	0.42	0.03/ (0- 0.07)†	0.67	0.83	0.01/ (0- 0.03)†
NSE	0.04	0.42	0.01/ (0- 0.04)†	0.55	0.83	0/ (0- 0.02)†
Extended model, 6-month mortality						
Composite (CB)	0.28	0.44	0.11/ (0.03- 0.22)†	0.76	0.76	0.06/ (0.01- 0.09)†
GFAP	0.11	0.39	0.02/ (0- 0.08)†	0.67	0.75	0.02/ (0- 0.03)†
NfL	0.14	0.39	0.08/ (0.02- 0.15)†	0.69	0.75	0.04/ (0.01- 0.06)†
Tau	0.13	0.39	0.05/ (0.01- 0.11)†	0.68	0.75	0.03/ (0- 0.05)†
UCH-L1	0.11	0.39	0.04/ (0.01- 0.09)†	0.66	0.75	0.02/ (0- 0.04)†
S100B	0.17	0.39	0.05/ (0.01- 0.1)†	0.70	0.75	0.01/ (-0.01- 0.04)
NSE	0.07	0.39	0.04/ (0- 0.11)†	0.58	0.75	0/ (-0.02- 0.02)

Note: Table presents 5 day biomarker TRAJ cluster IMPACT – Motor + GCS score model and IMPACT – Motor + GCS + Biomarker. Each individual biomarker group has 373 subjects, while the Composite (CB), 214. Bootstrap with R = 1000 iterations. † indicates the difference between models is significant.

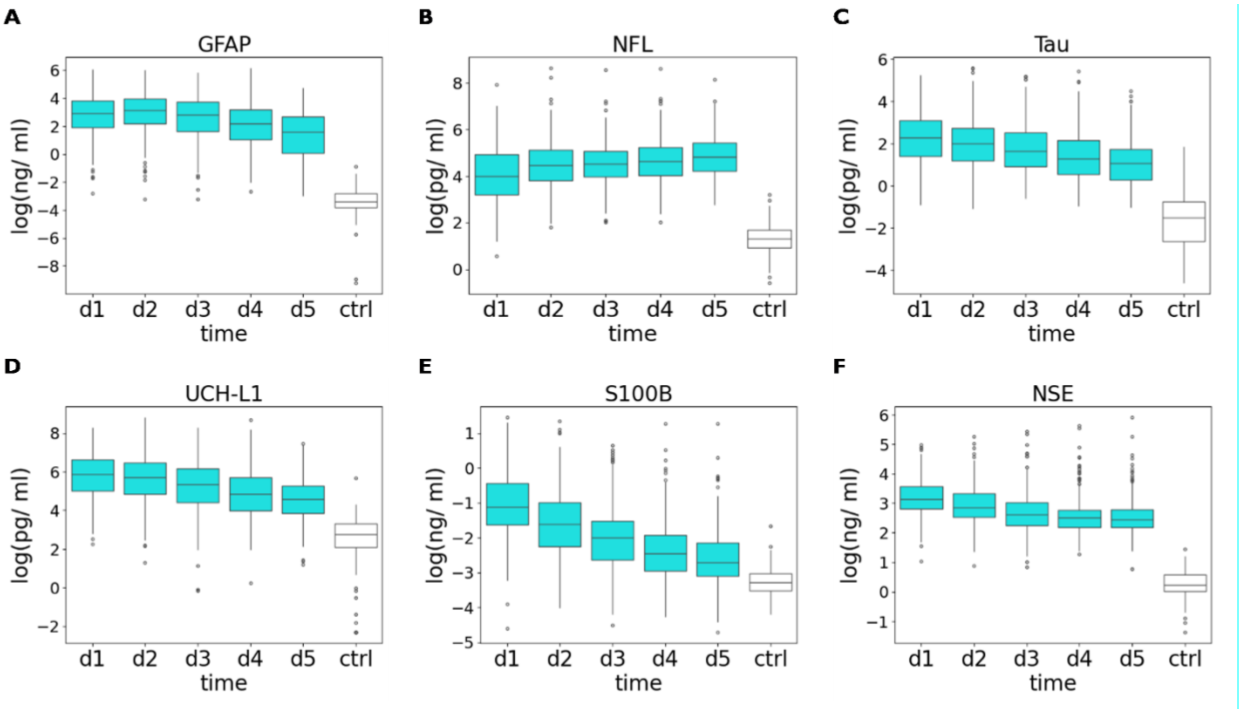

Supplementary Figure 1. Overall serum temporal Profile of all 6 TBI markers by median and IQR for TBI subjects, as compared to the levels of healthy controls. The TBI sample size for each biomarker is N=373 while for the controls, N=122 for biomarkers GFAP, NfL, Tau, and UCH-L1 and N=60 for NSE and S100B.

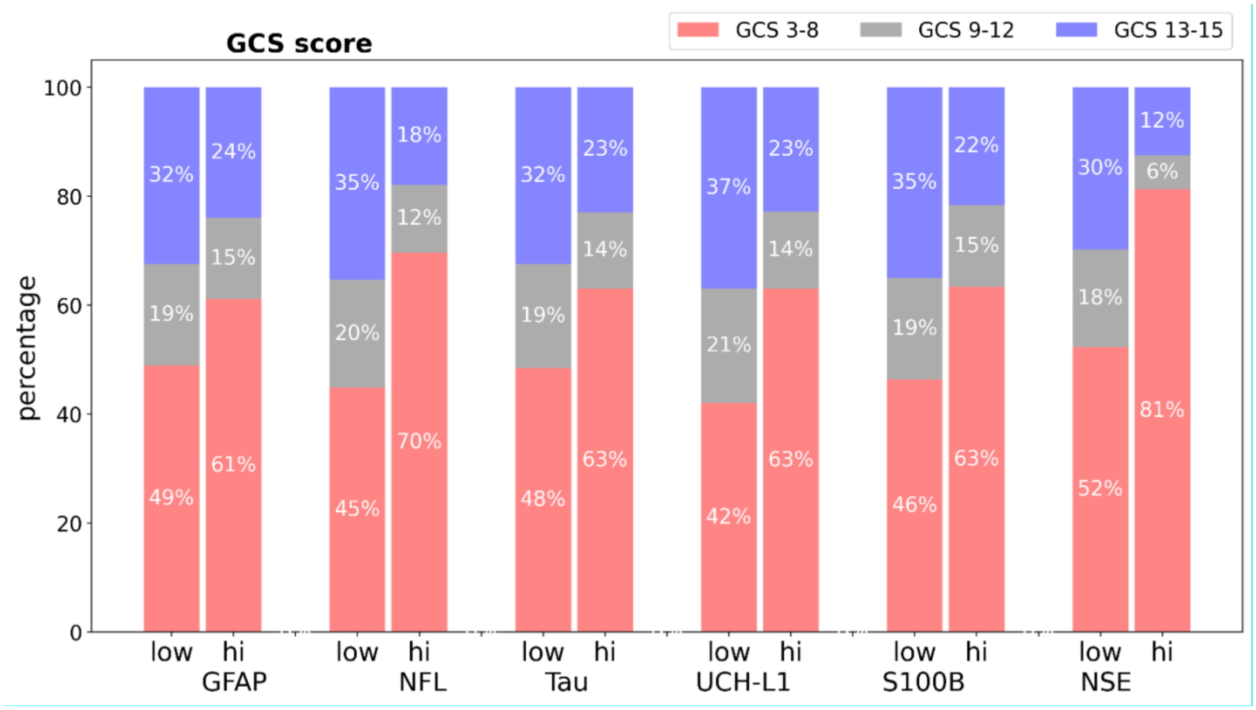

Supplementary Figure 2 Distribution of GCS score in low and high (hi) trajectory for each biomarker. GCS scores are categorized as: severe (3-8), moderate (9-12), mild (13-15). Group samples sizes: GFAP (Low: N=188, High: N=185), NfL (Low: N=220, High: N=153), Tau (Low: N=205, High: N=168), UCH-LI (Low: N=142, High: N=231), S100B (Low: N=181, High: N=192), and NSE (Low: N=339, High: N=34).

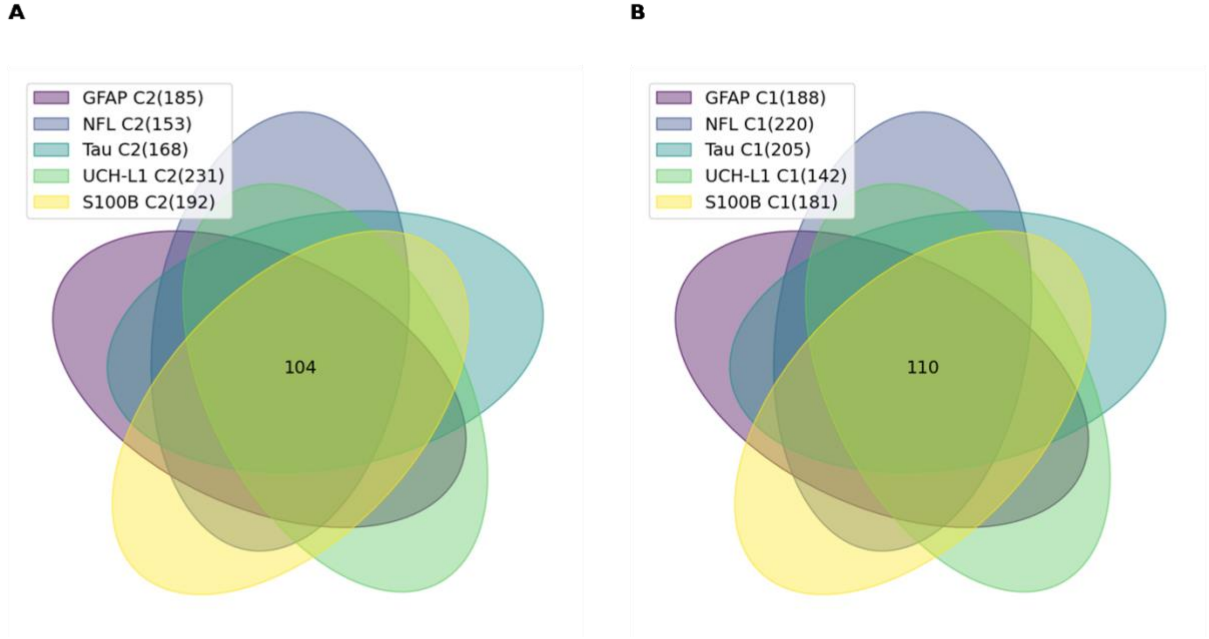

Supplementary Figure 3 Overlapping of 5 biomarkers low and high trajectory groups (GFAP, NFL, Tau, UCH-L1 and S100B). The center group for each diagram represent the composite biomarker (A) High TRAJ class (N=104) and (B) Low TRAJ class (N=110) respectively.

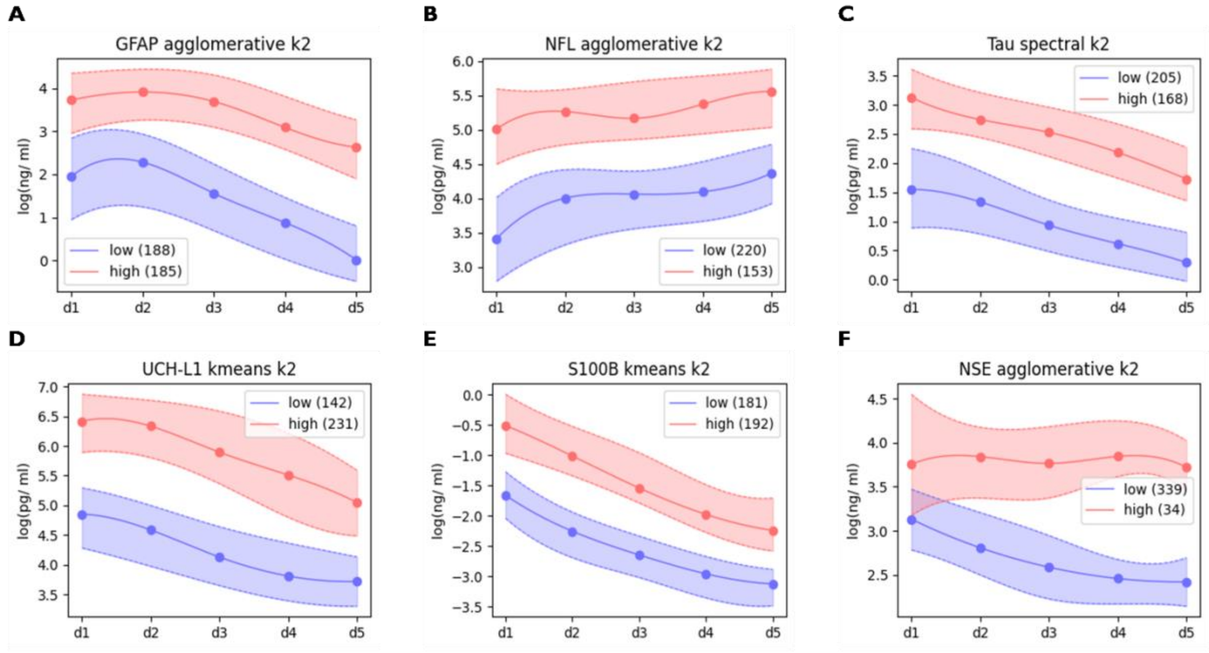

Supplementary Figure 4: Median and interquartile range of 5-day clusters in log scale from Day1 to Day 5. Note that the sample size for each group is specified in the legend.

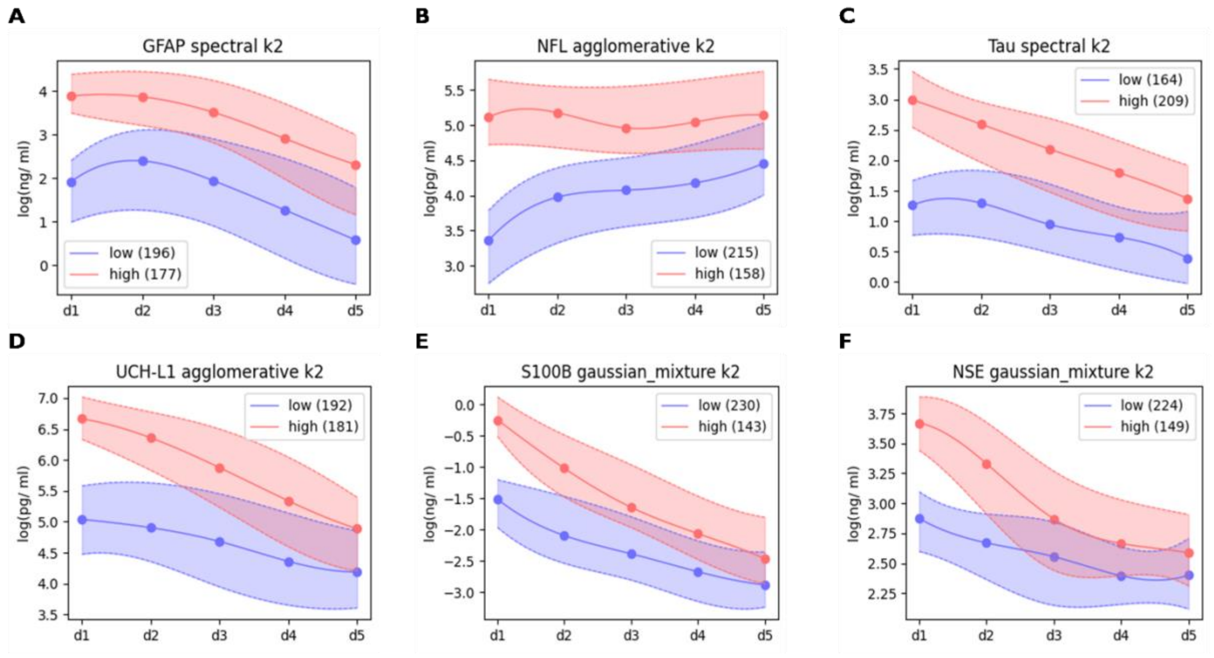

Supplementary Figure 5: Median and interquartile range of D1 clusters in log scale from day 1 to day 5. Note that the sample size for each group is specified in the legend.
